# IMU-Based Analysis of Postural Transitions: A Systematic Review

**DOI:** 10.3390/s26216808

**Published:** 2026-10-28

**Authors:** Riccardo Buraschi, Rosa Pullara, Cinzia Amici, Massimiliano Gobbo, Joel Pollet

**Affiliations:** 1 IRCCS Fondazione Don Carlo Gnocchi ETS, 20148 Milan, Italy; rburaschi@dongnocchi.it (R.B.);; 2 Department of Mechanical and Industrial Engineering, University of Brescia, 25123 Brescia, Italy; 3 Department of Clinical and Experimental Sciences, University of Brescia, 25123 Brescia, Italy

**Keywords:** postural transitions, IMU, movement analysis, rehabilitation

## Abstract

Background: Postural transitions (PTs) are crucial for daily independence, but their assessment often relies on subjective observational scales. Inertial measurement units (IMUs) offer quantitative assessment, yet a comprehensive synthesis of methodological approaches for IMU-based PT assessment is lacking. This systematic review maps the methodological landscape of IMU-based PT studies. Methods: A PCC-based search queried PubMed, Scopus, and Web of Science for IMU-based PT assessment in adults. Methodological quality was appraised using design- and purpose-appropriate established tools. Results: From 30,181 records, 35 studies were included, with heterogeneous study designs, purposes (e.g., detection, segmentation, validation), and analytical methods (e.g., threshold-based approach, model-based approach, deep learning method). Sit-to-stand was the most frequently assessed PT. Methodological appraisal indicated an intermediate robustness, with most studies showing moderate concerns. Sensor configurations, acquisition protocols, signal processing, segmentation, and outcome definitions varied substantially, and IMU-derived PT outcomes lacked standardization. Conclusion: IMUs offer significant potential for objective postural-transition assessment in rehabilitation, yet methodological heterogeneity and underrepresented tasks like bed mobility limit progress. Future research must prioritize standardized, validated, and clinically interpretable metrics to address reporting inconsistencies and establish a unified framework essential for widespread clinical adoption.

## 1. Introduction

Postural transitions (PTs) are essential motor tasks that allow individuals to move between functional positions such as lying, sitting, and standing, and therefore represent a fundamental prerequisite for independence in daily life [1,2,3]. These transitions are embedded in a wide range of everyday activities, including bed mobility, self-care, transfers, and mobility-related tasks, and are therefore highly relevant in rehabilitation settings. In people with disabling conditions, difficulties in performing PTs may compromise autonomy, reduce participation in daily activities, and increase dependence on caregivers. For these reasons, PT performance is highly relevant both as a rehabilitation target and as an indicator of functional recovery.

Importantly, PTs are not merely simple changes in body position. Their execution requires coordinated anticipatory and contemporary postural adjustments, trunk control, lower-limb force production, dynamic balance adaptation, and appropriate temporal organization of multi-segmental movements [4,5,6]. Because of this complexity, PTs can provide meaningful information about motor control and functional performance. In rehabilitation practice, a subject may complete a PT while still relying on compensatory strategies, altered movement sequencing, reduced smoothness, or inefficient postural control. Therefore, PT assessment should not be limited to whether the task is completed; it should also consider how it is performed. This distinction is particularly important when the clinical objective is to monitor functional recovery, to assess autonomy, or to identify persistent deficits [7].

Despite their clinical importance, routine assessment tools capture only part of PT. Commonly used clinical scales may include items related to transfer, balance, or mobility (e.g., Berg Balance Scale [8], items 1 and 4, and Functional Independence Measure [9], item I), which are useful for broad functional classification. However, these measures are predominantly observational, ordinal, and examiner-dependent, yielding gross functional outcomes, such as task completion or time to completion. As a result, subtle but meaningful aspects of the movement, including smoothness, fluidity, coordination, or symmetry, may remain unrevealed. This is particularly relevant when the aim is to monitor recovery, detect small changes over time, or distinguish compensation from more physiological motor improvement.

Within this context, wearable Inertial Measurement Units (IMUs) have emerged as promising tools for the quantitative assessment of PTs. Their use is particularly attractive in rehabilitation because they enable the integration of quantitative movement analysis with practical applicability in both clinical and potentially home-based settings [10,11]. By analyzing signals from onboard accelerometers and gyroscopes, IMUs provide a deeper and more detailed characterization of movement than standard observational assessment alone typically allows. More specifically, those devices may be used to derive quantitative descriptors of PT performance, including temporal and kinematic parameters that may reflect relevant aspects of movement quality [12]. They may also support the identification of key events within the transition and the segmentation of the movement into clinically or analytically meaningful phases [13]. In addition, they may enable the monitoring of PT-related behavior beyond structured testing sessions, thereby helping to bridge the gap between what a subject is able to do under standardized conditions and what the subject actually does in daily life.

The existing literature suggests that wearable inertial sensors are increasingly used to assess motor performance in rehabilitation and real-world settings. However, the available evidence syntheses have mostly addressed broader or adjacent topics rather than postural transitions (PTs) as a specific domain of investigation. Previous reviews have examined the use of wearable inertial sensors to quantify everyday-life motor activity in people with mobility impairments [14], the technological segmentation and analysis of the Instrumented Timed Up and Go Test [15], the application of wearable sensors to frailty assessment [16], static and dynamic balance assessment [17,18], and gait/posture characteristics related to fall risk [19]. More specifically, previous systematic reviews have focused on instrumented sit-to-stand and stand-to-sit assessments. Millor et al. [20] reviewed kinematic parameters derived from motion-sensor-based assessments of sit-to-stand and stand-to-sit transitions, whereas Shukla et al. [21] examined instrumented sit-to-stand assessments specifically for geriatric screening, including fall risk, frailty, and sarcopenia. These reviews were therefore primarily task- and/or population-specific. In contrast, the present review adopts a cross-cutting methodological perspective across multiple whole-body postural transitions, populations, and settings, integrating sensor configuration, acquisition protocols, signal processing and segmentation, analytical purpose and methods, outcome domains, and validation evidence. To the best of our knowledge, no systematic review has synthesized the methodological literature on IMU-based assessment of PTs across transition types, populations, sensor configurations, signal-processing pipelines, and data acquisition protocols. This leaves an important gap because PTs are clinically meaningful tasks, yet how they are instrumented, segmented, quantified, and classified remains scattered across heterogeneous lines of research. A focused methodological review is therefore necessary to clarify the current state of the art in the scientific literature.

Accordingly, this systematic review aims to map the methodological landscape of studies investigating PTs using inertial measurement units in healthy and pathological contexts. Specifically, the review examines and synthesizes how PTs have been instrumented, identified, and measured across the literature.

## 2. Methods

This systematic review followed the Cochrane Handbook (ver 6.4). The information reporting followed the PRISMA guidelines. The review protocol has been registered in the Open Science Framework (DOI of registration: 10.17605/OSF.IO/S52HV) [22].

### 2.1. Search Strategy and PCC Framework

The review question and eligibility criteria were structured according to the Population–Concept–Context (PCC) framework used. Population comprised healthy/non-clinical and clinical adult participants aged ≥18 years, without restriction to a specific clinical condition. Concept comprised IMU-based identification, detection, segmentation, classification, or quantitative assessment of whole-body postural transitions. Context included laboratory, clinical, and rehabilitation settings, home-like or free-living monitoring, and eligible secondary or benchmark datasets. MEDLINE (PubMed), Scopus, and Web of Science databases were queried for this review on 4 June 2026. Table 1 presents the complete search strategy. Two independent reviewers screened the records, blinded to each other (R.P. and R.B.). Possible disagreements were resolved by consulting a third reviewer (J.P.). The screening process was performed using Rayyan software [23], removing duplicates, then screening first by title and abstract, and finally by full text. In addition, further searches were conducted in the reference lists of the included articles (backward snowballing).

### 2.2. Eligibility Criteria

During screening and selection, we applied the following eligibility criteria. We considered studies written in English, involving healthy or diseased participants, adults, individuals of both sexes, with no publication-date restriction. The studies were required to use one or more IMU devices for the quantitative analysis, description, or identification of at least one postural transition. Postural transitions were defined as movements from one relatively stable body posture to another that involved a change in overall body configuration, as defined by Amici et al. [24]. Postural shifts, head movements, weight transfers, or arm lifts were not considered PTs and were therefore excluded from this review.

Studies relying exclusively on accelerometer-only devices were excluded. Static postural analyses, postural shifts, analyses of sports movements, or analyses of activities in the field of occupational medicine were not included. Furthermore, for selection, literature reviews, protocols, conference abstracts or proceedings, and theses were not considered.

### 2.3. Data Extraction

Two reviewers (R.P. and R.B.) worked independently and extracted and synthesized data by completing an Excel sheet. The data of interest were general information on the paper (author, year of publication, title, DOI, journal), study design, postural transition studied, any other movement considered, population of study (characteristics, number, age, sex), number of IMUs utilized and their specification (full scale range, bandwidth, sampling frequency, sensor positioning), any other device utilized, additional information about data acquisition setup and processing (algorithm, filter, cut-off frequency, software, signals), outputs, and a brief study description with results. The PTs considered were sit-to-stand, stand-to-sit, sit-to-supine, supine-to-sit, and roll. The transitions were also considered if part of a broader movement (e.g., lie to sit to stand to walk). The variables to be extracted were defined before data extraction. Given the heterogeneity of terminology and reporting across studies, conceptually similar extracted variables were subsequently grouped into descriptive categories for synthesis, including study design, analytical purpose, analytical method, population characteristics and sample size, sensor configuration and placement, acquisition protocol, and outcome domain. These categories were used for descriptive purposes and were not necessarily mutually exclusive. For each included study, we additionally extracted the reported quantitative performance of the IMU-based method, when available. Depending on study purpose, these included detection/classification accuracy, sensitivity, specificity, precision, recall, F1-score, AUC, timing error, agreement or criterion-validity measures, and reliability coefficients. Performance values were reported as provided by the original studies and were not converted into a common score. When method performance was not evaluated or not reported, entries were coded as N/A or NR, respectively. When available, the success rate was additionally reported as the percentage of correctly detected or classified events, activities, or subjects, using the percentage-based measure provided by the original study. Non-percentage performance metrics were not converted into success rates.

### 2.4. Appraisal of Studies’ Methodology

Methodological appraisal and quality assessment were performed immediately after data extraction for each included study, in accordance with the study design and analytical purpose. The adopted methodology integrates several tools to provide a comprehensive view of the included studies’ methodologies. The tools applied varied from those reported in the review protocol, as the initially programmed tools provided only a limited perspective on the methodological quality of the included studies. So, alongside the programmed tool, other tools were added. This constitutes a deviation from the preregistered appraisal plan and is reported transparently here. The Joanna Briggs Institute critical appraisal tools were applied to observational studies [25]. Quality In Prognosis Studies (QUIPS) tool was applied to prognostic studies [26]. The COnsensus-based Standards for the selection of health Measurement INstruments (COSMIN)-informed approach, adapted from the COSMIN Risk of Bias framework for studies on measurement properties, was applied to the studies evaluating measurement properties of wearable-sensor-derived PT outcomes [27,28]. When relevant, different appraisal tools were combined (e.g., JBI and COSMIN domains).

The COSMIN tool was adapted for this systematic review and applied only to specific, applicable domains, because most outcomes were sensor-derived or performance-based rather than patient-reported outcome measures.

No numerical score, weighting system, or quantitative cut-off was applied. An overall narrative judgment was assigned according to the number, severity, and likely impact of the identified methodological concerns on validity, interpretability, and applicability. The categories were “some concerns”, “moderate concerns”, “moderate-to-high concerns”, and “high concerns”. Supplementary Table S1 provides the applicability of each appraisal framework and the operational criteria used for the overall judgments. Methodological appraisal was not used as an eligibility criterion or to quantitatively weight study findings; rather, it informed the narrative interpretation of the strength and limitations of the available evidence.

## 3. Results

The database search identified 30,180 records across PubMed, Scopus, and Web of Science, and citation searching identified one additional record. After removing 3891 duplicates, we screened 26,289 records; we excluded 26,183 during title and abstract screening. Overall, 107 reports were sought for retrieval and assessed for eligibility, with all reports available for full-text assessment. Seventy-two reports were excluded because they did not assess postural transitions or did not use Inertial Measurement Units.

**Figure 1:**
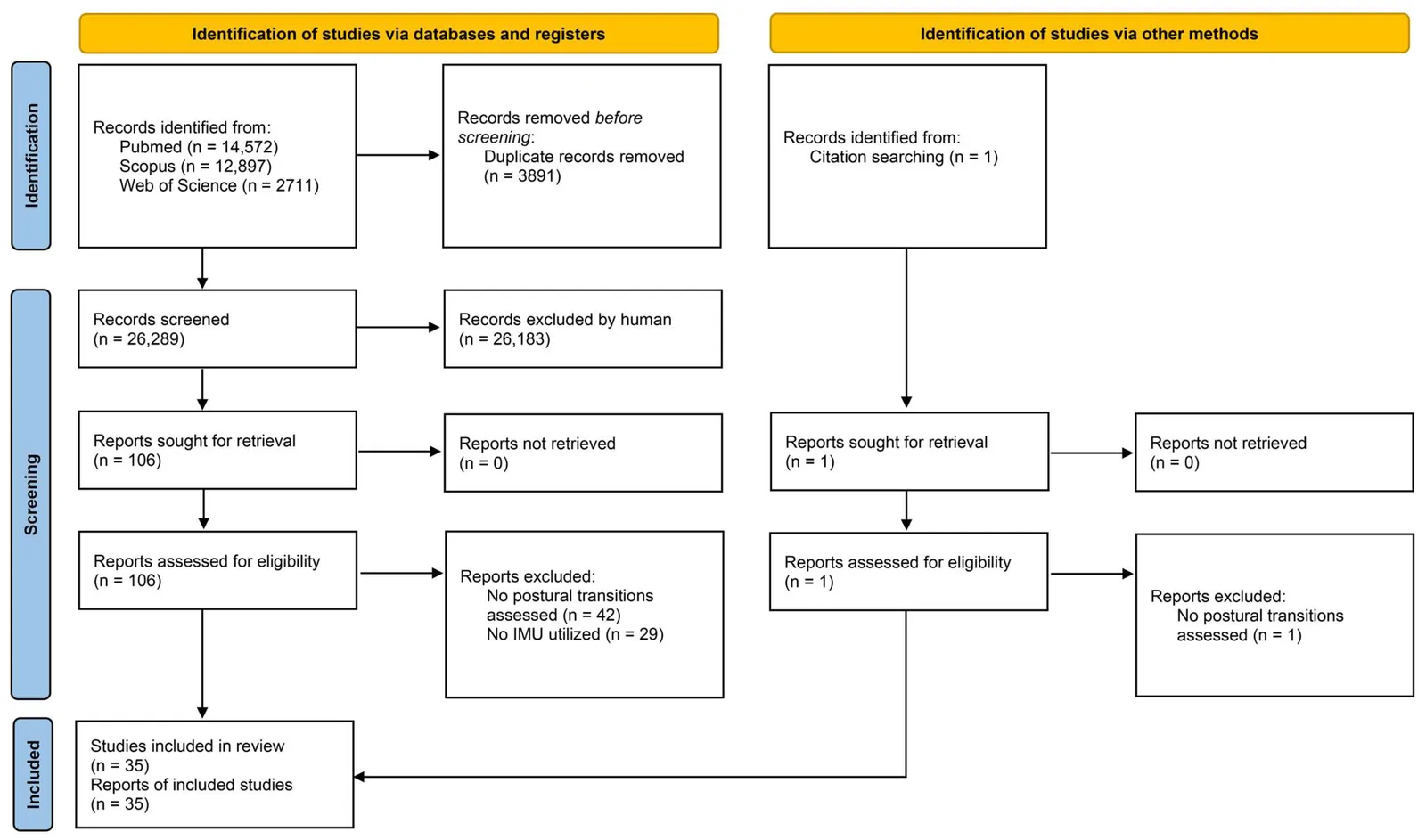
PRISMA flow diagram. This work is licensed under CC BY 4.0. To view a copy of this license, visit https://creativecommons.org/licenses/by/4.0/ (accessed on 25 October 2026).

One additional report was identified through backward citation searching and assessed for eligibility; it was excluded because it did not assess a postural transition according to the eligibility criteria. Finally, 35 studies were included [2,3,10,11,29,30,31,32,33,34,35,36,37,38,39,40,41,42,43,44,45,46,47,48,49,50,51,52,53,54,55,56,57,58,59] (Figure 1—PRISMA flow diagram and Supplementary Material S2, Table S2).

The included literature was methodologically heterogeneous. Study designs included cross-sectional observational or comparative studies, repeated measures and experimental studies, longitudinal/cohort studies, and secondary dataset analyses. Analytical purposes included PT detection and segmentation, quantification, classification, validation, and clinical association or discrimination. Analytical methods ranged from rule/threshold-based and biomechanical/model-based approaches to probabilistic models, conventional machine learning, and deep learning (Table 2).

### 3.1. Participants’ Characteristics and Study Settings

The included studies enrolled a broad range of populations, including healthy young and older adults, community-dwelling older adults, geriatric rehabilitation or hospital inpatients, and participants with neurological or other clinical conditions. Sample sizes varied considerably across studies. Among studies reporting participant-level samples, the smallest subgroup included five healthy volunteers [50], whereas the largest cohort included 1249 community-dwelling older adults [31]. However, several methodological and machine-learning studies reported data in terms of trials, acquisitions, windows, labeled samples, or public-dataset records, limiting direct comparison of overall sample sizes across the included literature.

Healthy or non-clinical participants were included either as the sole study population or as comparison groups, with ages ranging from young adult samples in their twenties to older adult and geriatric samples. Neurological populations were also frequently represented: studies included people with Parkinson’s disease [11,29,32,34,35,36,46,48,55], stroke [10,11,32,49,56], multiple sclerosis [10,11,32,57], and, in one study, Essential Tremor as a comparison group for early-stage Parkinson’s disease [34]. Parkinson’s disease samples included de novo or early-stage Parkinson’s disease, early-to-moderate idiopathic Parkinson’s disease, subgroups defined by Fear of Falling, medication state, dyskinesia, or postural-transition performance, and comparisons with healthy controls or Essential Tremor. Stroke samples included chronic stroke survivors able to walk independently, rehabilitation samples, participants with unilateral lower-limb motor dysfunction, and participants with stroke within mixed clinical or algorithm-validation cohorts. Multiple sclerosis was assessed in ambulatory participants and in heterogeneous pathological cohorts.

Other populations included frail or pre-frail older adults, older adults at high or low fall risk, participants with Locomotive Syndrome, geriatric inpatients with heart failure, acute-care hospital subjects, persons with unilateral transtibial amputation with or without low back pain, and mixed neurological rehabilitation cohorts. Age and sex were not reported uniformly. When reported, sex was generally described as male/female or women/men counts. Still, several studies or public datasets did not provide complete age and/or sex information, and some papers reported demographic characteristics only at the group level. Study settings were heterogeneous and included controlled laboratories, rehabilitation clinics, hospital wards, community or residential-care environments, free-living daily activity monitoring, and public benchmark datasets. Characteristics of the included studies are presented in Table 2.

### 3.2. Appraisal of the Included Studies’ Methodology

Methodological appraisal showed a heterogeneous but overall intermediate level of methodological robustness across the included studies, as reported in Table 3 and defined in Supplementary Material S1, Table S1. Most studies were classified as having moderate concerns (23 out of 35), indicating limitations that did not seriously compromise the interpretation of the main findings. Four studies showed some concerns [45,47,50,58], whereas eight were classified as having moderate-to-high concerns [34,39,40,49,51,52,54,56]. No study was classified as having high concerns. Recurrent methodological issues included small sample sizes, heterogeneous or non-clinical samples, incomplete control of confounding, and limited participant-level reporting. In clinical-observational studies, concerns mainly relate to exploratory designs and the lack of reliability, measurement error, or agreement analyses for IMU-derived outcomes [10,32,57]. In validation and algorithm-development studies, common limitations included the absence of external biomechanical reference standards and limited event-level validation of PT onset, offset, duration, or segmentation [46,48]. In machine-learning and deep-learning studies, concerns mainly related to public or secondary datasets, window-level activity classification rather than PT-specific segmentation, limited external validation, class imbalance, and potential overfitting [41,49,51,52]. Detailed methodology appraisal is presented in Table 3.

### 3.3. Postural Transitions Assessed

Sit-to-stand transition was assessed in all 35 included studies, whereas stand-to-sit was reported in 25 studies [3,10,11,29,31,32,33,34,37,38,39,40,42,43,44,45,46,47,48,51,52,55,56,57,59]. Transitions involving lying or bed mobility were less frequently investigated, including supine- or lying-to-sit [2,10,45], sit-to-supine or sitting-to-lying [10,45], and rolling or turning in bed [10,45,52]. Table 4 reports the details of the PTs assessed within the studies.

### 3.4. Setup and Acquisition Protocols

Sensor configurations varied substantially across the included studies, ranging from single body-worn sensors to multi-sensor full-body or lower-limb configurations. Single-sensor approaches were common, particularly in studies focused on clinical feasibility, daily-life monitoring, or simplified postural-transition assessment, with sensors most frequently positioned on the trunk, including the sternum, thorax, lower back, lumbar region, waist, pelvis, or hip [2,3,10,31,32,33,35,39,40,45,46,47,58]. Other single-sensor placements included the thigh, waist, hip, and pocket positions, particularly in activity-recognition or probabilistic classification studies [43,51]. Several studies used multi-sensor configurations to capture lower-limb and trunk kinematics, including two-sensor setups on the thigh and trunk, shank and back, upper trunk and sacrum, or thigh and lumbar region; three-sensor configurations on the lower back and feet, ankle/thigh/wrist, or trunk/pelvis positions; and larger systems using four, six, seven, eight, or ten IMUs [11,29,30,34,37,38,41,42,44,48,50,53,54,55,56,57,59]. Multi-sensor setups were mainly used when the objective was to reconstruct segmental kinematics, analyze instrumented clinical tests in detail, or support phase classification models [34,41,42,48,49,50,55,56,57]. A subset of studies also combined IMUs with non-IMU systems, including force plates, optical motion-capture systems, chair switches, video recordings, pressure-sensor insoles, depth-camera skeletal tracking, or surface electromyography [11,39,41,42,44,46,47,48,50,55,57]. Sampling frequency was inconsistently reported but, when available, ranged from very low-frequency wearable acquisition to commonly used rates of 40–200 Hz [33,39,45,50]. Public-dataset and machine-learning studies often used dataset-specific acquisition settings, whereas clinical and rehabilitation studies generally used standardized tasks such as the Timed Up and Go Test, repeated sit-to-stand tests, five-times sit-to-stand, structured mobility batteries, or customized postural-transition protocols [10,30,32,40,51,52,53,54,56,57,58]. Acquisition settings included laboratory environments, rehabilitation clinics, hospital wards, community settings, home-like environments, free-living daily-life monitoring, and public benchmark datasets [2,3,11,29,30,31,39,46,50,53,54]; see Table 5.

### 3.5. Signal Processing and Segmentation Approaches

The extracted signal-processing pipelines showed marked heterogeneity. The most commonly used raw signals were triaxial acceleration and angular velocity, but several studies also used magnetometer signals, orientation estimates, Euler angles, quaternions, trunk pitch, vertical acceleration, angular displacement, linear acceleration after gravity removal, joint angular kinematics, or derived global-frame quantities [2,10,11,30,31,34,40,41,45,47,49,50,55,57,58]. Some studies explicitly decomposed acceleration into gravitational and body-motion components, transformed local sensor acceleration into a global coordinate frame, or used sensor-fusion procedures to estimate orientation [2,10,11,35,46,48,50,58]. Filtering was not uniformly reported. When described, processing included low-pass, high-pass, band-pass, or notch filtering; Butterworth filters with cut-off frequencies ranging from 0.2 Hz to 25 Hz; zero-phase filtering; discrete or continuous wavelet transforms; Kalman or extended Kalman filtering; quaternion-based orientation estimation; moving-average smoothing; normalization; resampling; and window-based preprocessing [2,10,11,37,38,40,45,46,47,48,50,51,52,54,57,58]. Segmentation methods also differed considerably. Some studies used threshold-based algorithms to detect movement onset and offset from acceleration, angular velocity, trunk tilt, pitch angle, femoral acceleration, or signal variance [2,10,36,38,44,49,50,56,58]. Others used more-complex event-detection models, including continuous wavelet transform peak detection followed by sigmoid fitting, discrete wavelet transform combined with kinematic rules, trunk-tilt peak detection, yaw/pitch angular-velocity rules for TUG phase segmentation, chair-switch-based event prediction, or probabilistic state modeling [11,39,43,45,47,55]. Several studies relied on manual segmentation, commercial software outputs, investigator-generated labels, video-based labels, or dataset-provided annotations [34,42,51,52,53,54,56]. Machine-learning studies generally segmented continuous signals into fixed-length overlapping windows, whereas phase-recognition studies used manually labeled or event-defined sit-to-stand phases as model targets [41,49,51,52,54]. Reporting of technical acquisition and processing procedures was heterogeneous. Only a few studies explicitly described sensor-to-segment or anatomical alignment, whereas transformations to anatomical or global coordinate systems were reported more frequently [2,11,34,35,41,50,58]. Studies explicitly described synchronization with external reference systems using force plates, chair switches, or optical motion-capture systems [41,47,48,50], while information on calibration procedures and data loss or exclusion was less consistently available. Several studies reported sensor-fusion or orientation-estimation procedures, including Kalman-filter- and quaternion-based approaches [2,11,35,46,48,50,58]. Definitions of transition onset, offset, and subphase boundaries varied considerably across studies, ranging from acceleration- or angular-velocity-based thresholds to wavelet-based, probabilistic, reference-system-based, and machine-learning approaches. Overall, only a subset of studies provided sufficiently detailed information on filtering, event definitions, and segmentation procedures to reproduce the analytical pipeline [2,10,38,40,41,45,46,47,48,50,57].

### 3.6. Detection and Classification Algorithms

The analytical approaches could be grouped into rule-based detection, model-based segmentation, probabilistic classification, conventional machine learning, and deep-learning approaches. Rule-based or threshold-based algorithms were used to detect task start/end points, sit-to-stand and stand-to-sit events, TUG subphases, trunk movements, or angular-velocity thresholds [2,10,36,38,39,40,45,50,55,56]. Other studies used biomechanically informed models, such as trunk-tilt algorithms, wavelet-based detection, vertical-displacement estimation, sigmoid fitting, weighted event-prediction models, or three-segment kinematic reconstruction [11,45,46,47,48,50]. Probabilistic methods were also represented, including Bayesian evidence accumulation for sit, transition, and stand states [43]. Machine-learning and deep-learning approaches were mainly used in two contexts: Human-Activity-Recognition and postural-transition phase classification. Conventional classifiers included Random Forests, support vector machines, dendrogram-based SVMs, logistic regression, k-means clustering, and ensemble approaches [3,35,37,40,44,51]. Deep-learning models included CNN–BiLSTM–Attention networks, LSTM classifiers, parallel CNN–Transformer architectures, multi-branch convolutional neural networks, stacking ensembles, and Transformer-based dual-task learning models [41,42,49,51,52,54]. However, the purpose of classification differed across studies. Some algorithms specifically targeted postural-transition detection, direction classification, or phase recognition, whereas others treated sit-to-stand, stand-to-sit, lying, sitting, and standing as activity classes within broader Human Activity Recognition datasets [11,41,42,43,44,49,51,52,54]. Therefore, high classification performance in HAR studies should not be interpreted as direct evidence of accurate biomechanical segmentation of postural-transition onset, offset, or clinically meaningful subphases [51,52,54]. Accordingly, algorithm performance should be interpreted in relation to its intended analytical purpose and validation strategy. High classification accuracy in HAR or posture-recognition tasks should not be considered equivalent to accurate biomechanical segmentation or valid quantitative assessment of postural transitions.

### 3.7. IMU-Derived Outcome Domains

The extracted outcomes covered several domains. Temporal parameters were the most frequently reported outputs and included total TUG time, sit-to-stand duration, stand-to-sit duration, transition duration, phase duration, seat-off and seat-on timing, time-to-completion, number of transitions, transition counts per hour, duration of daily activity states, and model response or inference time [2,3,11,29,30,31,32,33,37,39,40,42,45,46,47,53,55,56,57,58]. Kinematic parameters were also widely reported and included trunk or segmental acceleration, angular velocity, angular displacement, trunk tilt, pitch/yaw/roll angles, Range of Motion, peak angular velocity, vertical displacement, gait-related parameters, turning metrics, joint angles, and reconstructed shank, thigh, back, hip, knee, ankle, or pelvis kinematics [2,11,30,31,34,35,36,37,39,40,41,44,45,47,48,49,50,55,56,57,58,59]. Several studies extracted movement-quality parameters, including jerk, RMS jerk, smoothness, fluency, fractal dimension, wavelet energy, movement irregularity, acceleration variability, and trunk movement variation [2,10,30,34,38,39,57,58] A smaller group of studies focused on force-, power-, or energy-related outcomes, such as peak power during sit-to-stand, force-related trunk estimates, vertical force or power derived from acceleration, kinetic energy, and Ground-Reaction-Force-based phase labeling [11,29,35,41,49,50,58]. Classification-oriented studies reported accuracy, precision, recall, F1-score, sensitivity, specificity, AUC, confusion matrices, response time, cross-entropy loss, or inference time [40,41,42,43,44,48,49,51,52,53,54]. Overall, the literature moved beyond simple task duration, but there was no standardized set of IMU-derived postural-transition outcomes. The same construct was often operationalized differently across studies, particularly for smoothness, fluency, power, and phase-specific kinematic descriptors [2,10,11,30,35,47,50,57,58].

### 3.8. Reference Standards, Reliability, and Validity Evidence

Reference standards and validation procedures were heterogeneous and often incomplete. Some studies used external biomechanical or observational references, including optical motion capture, force plates, chair switches, video recordings, stopwatch timing, observer labels, depth-camera skeletal tracking, Codamotion, or Optotrak systems [11,39,41,42,46,47,48,50,55,57]. Others relied on dataset-provided labels, manual annotations, investigator-generated timestamps, commercial software outputs, or clinical group membership as reference information [32,34,51,52,53,54,56]. In several clinical studies, IMU-derived parameters were related to clinical measures or diagnostic groupings, such as balance, disability, frailty, Fear of Falling, Locomotive Syndrome, fall-risk classification, heart-failure severity, Parkinson’s disease severity, or multiple-sclerosis-related disability [3,11,30,32,34,35,37,39,57]. Reliability and validity evidence varied according to study purpose. Test–retest reliability was specifically addressed in a limited number of studies, including analyses using ICCs, SEM, repeated assessments, or the repeatability of TUG- or sit-to-stand-derived features [55,57,58]. Some studies assessed agreement or criterion validity using errors relative to chair switches, force plates, optical motion capture, stopwatch timing, video, or observer records [41,42,47,48,50,55,57]. Several algorithmic and machine-learning studies reported internal performance metrics, cross-validation, train-test splits, or independent test-set results, but did not provide reliability, measurement error, or agreement analyses for PT-specific onset, offset, duration, or subphase boundaries [39,41,42,43,51,52,54]. Therefore, classification performance metrics were considered separately from evidence of criterion or concurrent validity, reliability, agreement, clinical discriminative ability, and ecological validity. Thus, while many studies demonstrated promising classification or discrimination performance, the strength of evidence for reproducible and externally validated postural-transition measurement remains uneven. Table 6 summarizes study-level performance, success rate, and validation results, along with sensor configuration and reference standard. Reported performance varied substantially across analytical purposes and validation contexts, and direct comparisons between methods or sensor configurations were therefore not considered appropriate.

### 3.9. Clinical and Functional Interpretation of Postural-Transition Metrics

Researchers used IMU-derived postural-transition metrics to characterize functional limitations across several populations. In stroke survivors, instrumented clinical tests captured slower task performance and dual-task-related changes in TUG, sit-to-stand, walking, and balance-related measures [10,56]. In Parkinson’s disease, researchers used postural-transition and TUG-related metrics to characterize Fear of Falling, early disease versus Essential Tremor, sit-to-stand performance, turn-to-sit behavior, and phase-specific movement abnormalities [29,32,34,35,36,46,48,55]. Relevant parameters included transition duration, peak angular velocity, trunk or limb kinematics, phase-recognition outputs, and combined laboratory and daily-life mobility features [29,34,35,46,48,55]. In multiple sclerosis, thigh-derived angular-velocity features during sit-to-stand and stand-to-sit were reported as clinically informative and more discriminatory than some purely temporal measures [57]. In older adults, frail individuals, and fall-risk populations, transition metrics described longer transition durations, altered smoothness, reduced daily transitions, cautious sitting behavior, fall-risk-related TUG abnormalities, and associations with adverse health outcomes [2,3,31,33,39,40,50,58]. Other applications included geriatric inpatients with heart failure, individuals with Locomotive Syndrome, persons with transtibial amputation with or without low back pain, acute-care hospital subjects, and mixed rehabilitation cohorts [10,30,37,38,53]. Across these populations, the most clinically interpretable findings generally came from studies linking IMU-derived features to functional status, clinical scales, group discrimination, frailty or fall-risk categories, or longitudinal health outcomes [3,29,30,31,32,33,34,35,37,39,40,57]. However, the clinical meaning of many algorithmic or high-dimensional features remained less direct, especially when extracted primarily for activity recognition rather than for PT-specific biomechanical assessment [49,51,52,54]. The main IMU-derived outcome domains and their clinical or functional interpretation across the included studies are summarized in Table 7.

The full detailed data extracted from the included studies are presented in Supplementary Material S3, Table S3.

## 4. Discussion

This systematic review mapped the methodological landscape of IMU-based assessment of postural transitions and identified three main findings. First, the literature is heavily focused on sit-to-stand and transitions embedded within the Timed Up and Go Test. In contrast, stand-to-sit and, especially, bed-mobility transitions (e.g., supine-to-sit and rolling) remain underrepresented. Second, substantial heterogeneity exists across sensor configurations, acquisition protocols, signal-processing pipelines, segmentation rules, and outcome definitions. Third, although many studies reported promising discrimination, classification, or clinical associations, evidence supporting the reproducibility, measurement error, criterion validity, and external validity of IMU-derived postural-transition measures remains uneven. Taken together, these findings indicate that IMUs have clear potential to extend postural-transition assessment beyond observational scales and total task time, but the field has not yet converged on sufficiently standardized and clinically interpretable measurement approaches. However, the results of these reviews also showed a focus on specific pathological populations (e.g., neurological disorders or older adults), neglecting other populations that could benefit from specific studies in the field (e.g., subjects with rheumatic diseases or younger populations). The distribution of the included populations also revealed a clinically relevant evidence gap. Research was concentrated primarily in neurological populations, older adults, frailty or fall-risk cohorts, and selected rehabilitation populations, whereas other clinical groups in which postural transitions may also represent an important functional limitation, such as rheumatological populations, were absent or poorly represented. Because no diagnosis-specific eligibility restriction was applied, this pattern reflects the current distribution of the available evidence rather than a restriction imposed by the review. These findings extend adjacent reviews of everyday motor activity, instrumented TUG, frailty, balance, and fall-risk assessment [14,15,16,17,18,19] by showing that, when postural transitions are considered as a distinct methodological domain, heterogeneity extends from hardware and acquisition protocols to event definitions, outcome constructs, and measurement-property evidence.

Study-level performance data indicate that high performance can be achieved with both single- and multi-sensor configurations. However, differences in populations, tasks, reference standards, outcome definitions, and validation strategies preclude ranking algorithms or identifying an optimal number of sensors based on performance percentages alone. Sensor configuration should therefore be interpreted in relation to the intended application and validation context.

The marked predominance of sit-to-stand is understandable because chair rise is frequent in daily life, is easy to standardize, and challenges lower-limb force production, trunk control, balance, and movement coordination [1,4,5,6]. The predominance of sit-to-stand and TUG-related transitions may also reflect their incorporation into widely used standardized clinical mobility assessments, which makes these transitions relatively easy to administer and instrument compared with more complex functional transitions. However, this focus means that the available evidence covers only a limited range of postural transitions. Only a small minority of the included studies investigated transitions involving supine-to-sit, sit-to-supine, and rolling [2,10,45,52]. These tasks are particularly relevant in geriatric and neurologic rehabilitation because rolling and supine-to-sit are components of basic postural control and functional mobility [60]. The current evidence base therefore represents only part of the postural-transition continuum and may underrepresent the needs of people with more-severe disability, who are often unable to perform a conventional TUG or repeated chair-rise protocol.

Sensor configuration reflected a recurring trade-off between feasibility and biomechanical detail. Single-sensor approaches, most commonly located on the sternum, lumbar region, lower back, waist, or pelvis, were widely used in studies prioritizing clinical practicality, unobtrusive monitoring, or free-living assessment [2,3,10,11,31,32,33,35,39,40,58]. A trunk-mounted IMU can capture global orientation, angular velocity, acceleration, and transition timing with minimal setup burden, making this configuration attractive for routine clinical use and remote monitoring. Conversely, multi-sensor systems were generally adopted when the aim was to reconstruct segmental kinematics, distinguish transition phases, quantify lower-limb behavior, or support exoskeleton control [11,34,37,41,42,48,49,50,55,56,57,59]. These systems provide richer biomechanical information but increase preparation time, calibration requirements, data-processing complexity, and potential sensitivity of the underlying biomechanical model to placement and sensor-to-segment alignment errors [61]. Future studies should therefore justify sensor number and placement in relation to the intended clinical construct, rather than assuming that a larger sensor set is inherently superior.

Protocol heterogeneity further limited comparability. Postural transitions were examined as isolated movements, repeated chair-rise tasks, components of TUG protocols with different procedures, parts of longer lie-to-sit-to-stand-to-walk sequences, events observed during hospital admission or daily life, and activity classes within public Human-Activity-Recognition datasets [2,3,11,29,30,34,40,45,51,52,53,54,59]. These paradigms do not measure the same phenomenon. Standardized laboratory tasks primarily characterize motor capacity under controlled conditions, whereas free-living monitoring captures habitual performance in everyday contexts; these constructs are related but should not be treated as interchangeable [60]. Similarly, an activity-recognition dataset may be suitable for distinguishing broad movement classes but may not contain sufficiently precise labels for biomechanical onset, offset, seat-off, seat-on, or clinically meaningful subphases. This distinction should be made explicit because performance observed during a structured test cannot be assumed to represent spontaneous behavior in everyday life, and high activity-classification accuracy cannot be interpreted as evidence of accurate transition segmentation.

Signal processing and segmentation were among the most heterogeneous and incompletely reported methodological areas. In particular, reporting of coordinate-system transformations, sensor alignment and calibration, synchronization procedures, and transition event definitions was inconsistent across studies. This reporting gap is not merely technical, because differences in coordinate processing, filtering, and event definitions can alter kinematic estimates, while derivative-based outcomes such as jerk and smoothness are particularly sensitive to signal-processing and computational choices [61,62]. Incomplete reporting of these procedures therefore limits reproducibility and may make it difficult to compare nominally similar IMU-derived outcomes across studies.

The increasing use of machine-learning and deep-learning methods expands the potential for automatic monitoring, but also requires careful interpretation. Conventional classifiers and neural-network architectures achieved encouraging results for activity recognition, posture-state identification, or sit-to-stand phase classification [3,35,37,40,41,42,43,44,49,51,52,54]. Nevertheless, model performance was frequently reported at the window or activity-class level, sometimes using public or secondary datasets, and was not always accompanied by event-level errors for transition onset, offset, duration, or phase boundaries. Validation strategies in machine-learning studies were heterogeneous and included cross-validation, random train–test splits, hold-out testing, and participant- or window-level evaluation. Participant-independent and external validation were not consistently demonstrated, and in some studies the available reporting did not allow the independence of training and test data at the participant level to be established clearly, a relevant issue because inadequate data partitioning may lead to optimistic performance estimates and data leakage [63,64]. Class imbalance, small transition-class samples, and potential overfitting were also identified as methodological concerns in some studies. These differences limit direct comparison of reported classification performance and should be considered when interpreting apparently high accuracy values.

For clinical use, algorithms should be tested on data from participants not used for training, with a clear separation between training, validation, and test sets, and with measures that reflect their intended purpose. For example, a model designed to count daily transitions should report missed and falsely detected events, whereas a model designed to identify movement phases should report timing errors against a synchronized reference.

Temporal outcomes were the most common and clinically accessible measures, including total task time, transition duration, phase duration, seat-off or seat-on timing, and daily transition counts. However, one of the main advantages of IMU-based assessment is its ability to characterize how a transition is performed, rather than only whether or how quickly it is completed. Several studies therefore examined angular velocity, trunk inclination, acceleration, movement variability, smoothness, fluency, jerk, power, or reconstructed segmental kinematics [2,10,11,30,33,34,35,36,37,38,39,47,48,50,57,58]. These measures may reveal compensatory strategies or impaired motor control that are not captured by total time alone. Inconsistent definitions and computational procedures nevertheless constrain their clinical use. Moreover, an instrumented measure is not necessarily more clinically meaningful or responsive than a functional assessment, as rehabilitation-related improvements detected by functional scales may not be effectively mirrored by instrumented balance measures [65]. Evidence regarding reliability, measurement error, agreement, criterion validity, responsiveness, and external validation remained limited or inconsistently reported across the included studies. These measurement properties therefore represent important priorities for future validation of IMU-derived postural-transition outcomes.

Before adopting IMU-derived transition outcomes as clinical endpoints, studies should verify the sensor signal, analytically validate the algorithm-derived metric, and clinically validate it within a defined population and context of use, along with reliability, measurement error, criterion or construct validity, responsiveness, and interpretability [66].

The methodological appraisal supports a cautious interpretation of the evidence. Most studies had moderate concerns, with recurrent limitations related to small or selected samples, exploratory designs, incomplete control of confounding, limited participant-level reporting, lack of external reference standards, and insufficient validation in independent clinical cohorts. No study was classified as having great methodological concerns, suggesting that the current literature provides a credible basis for continued development; however, the predominance of intermediate-quality evidence means that reported performance should not be considered definitive.

The most recurrent reporting gaps concerned the operational definition of postural transitions and their phases, sensor configuration and placement, signal-processing procedures, event-detection rules, and validation strategy. More-complete reporting of these elements would improve reproducibility and facilitate cross-study comparisons.

To our knowledge, no previous review has specifically integrated multiple whole-body postural transitions across populations and settings while simultaneously mapping sensor configuration, acquisition protocols, signal-processing and segmentation approaches, analytical methods, outcome domains, and validation evidence. This review therefore treats postural transitions as the unit of methodological synthesis and follows the complete measurement pathway from sensor configuration and acquisition to processing, segmentation, derived outcomes, and validation.

This review has several limitations. The broad inclusion of clinical, observational, algorithm-development, validation, and Human-Activity-Recognition studies was necessary to map the field comprehensively, but it also increased conceptual and methodological heterogeneity and precluded meta-analysis. The review focused on systems including an IMU and excluded accelerometer-only studies, so it may not have captured some relevant activity-monitoring literature. We excluded conference proceedings, abstracts, protocols, and theses, which may have reduced the coverage of rapidly evolving engineering methods. Finally, because no single appraisal tool fully addresses the diversity of sensor-based methodological studies, we evaluated study quality using design-appropriate tools and an adapted, narrative integration of applicable domains.

## 5. Conclusions

IMU-based analysis provides a feasible approach for quantifying postural transitions across laboratory, clinical, and free-living settings, but the available evidence is strongly concentrated on sit-to-stand and TUG-related tasks. Bed-mobility transitions, particularly supine-to-sit, sit-to-supine, and rolling, remain markedly underrepresented. The main methodological gaps identified by this review concern inconsistent definitions of transitions and event boundaries, heterogeneous acquisition and processing procedures, and limited evidence regarding reliability, measurement error, criterion validity, and external validation. Future research should therefore prioritize reproducible transition definitions and processing methods, stronger evaluation of measurement properties, participant-independent and real-world validation, and investigation of postural transitions and clinical populations that are currently underrepresented. Addressing these gaps is necessary for IMU-derived measures to become reproducible and clinically interpretable tools for rehabilitation assessment and monitoring.

## Figures and Tables

**Table 1:** Search strategy.

Database	Search Strategy
MEDLINE (PubMed)	1. (postural balance[MeSH Terms]) OR (sitting posture[MeSH Terms]) OR (standing posture[MeSH Terms]) OR (supine posture[MeSH Terms]) OR (movement[MeSH Terms]) 2. “postural transition”[Title/Abstract] OR “sit”[Title/Abstract] OR “sitting”[Title/Abstract] OR “stand”[Title/Abstract] OR “roll”[Title/Abstract] OR “supine”[Title/Abstract] OR “lying”[Title/Abstract] OR “standing”[Title/Abstract] 3. #1 OR #2 4. (wearable electronic device[MeSH Terms]) OR (accelerometry[MeSH Terms]) OR (magnetometry[MeSH Terms]) 5. (gyroscope[Title/Abstract]) OR (IMU[Title/Abstract]) OR (inclinometer[Title/Abstract]) OR (accelerometer[Title/Abstract]) OR (“inertial measurement unit”[Title/Abstract]) 6. #4 OR #5 7. “humans”[MeSH Terms] 8. “adult”[MeSH Terms] OR “adult”[Title/Abstract] 9. #3 AND #6 AND #7 AND #8
Scopus	1. ((“functional movement” OR “postural transition” OR “sit” OR “sitting” OR “stand” OR “roll” OR “supine” OR “lying” OR “standing”) AND (“sensor” OR “gyroscope” OR “IMU” OR “inclinometer” OR “accelerometer” OR “inertial measurement unit”) AND “human”)[ti, ab, kw] 2. Filter: Article
Web of Science	1. TS = (“functional movement” OR “postural transition” OR “sit” OR “sitting” OR “stand” OR “roll” OR “supine” OR “lying” OR “standing”) 2. TS = (“sensor” OR “gyroscope” OR “IMU” OR “inclinometer” OR “accelerometer” OR “inertial measurement unit”) 3. TS = (human) 4. #1 AND #2 AND #3

**Table 2:** Characteristics of the included studies.

Study	Study Design	Analytical Purpose	Analytical Method	Population	Sample Size	Clinical Condition/Group	Setting	Task/Test	Postural Transitions Assessed
Abdollahi et al. (2024) [56]	Cross-sectional mixed-factorial repeated-measures study,	Quantification; group and dual-task comparison,	Threshold-based event detection + descriptive/inferential statistics,	Stroke survivors and healthy controls,	N = 41; stroke survivors n = 21; healthy controls n = 20,	Stroke survivors at least 6 months post-stroke with independent walking ability over 10 m; healthy controls,	N/A,	TUG, five-repetition STS, 10MWT, and 30 s balance tests with eyes open/closed, each under single-task and dual-task conditions,	Sit-to-stand; Stand-to-sit,
Abraha et al. (2026) [51]	Secondary dataset analysis,	Classification/recognition of PT and activity classes.	Conventional machine learning: Stacking ensemble (Random Forest + XGBoost + logistic-regression meta-learner).	Participants from three public benchmark HAR datasets: KU-HAR, UCI-HAR, and USC-HAD.	KU-HAR: 90 participants, 1945 raw samples and 20,750 windowed segments; UCI-HAR: 30 volunteers and 10,299 labeled samples; USC-HAD: 14 participants and 840 trials.	No clinical condition/group reported; benchmark datasets of daily activities and transitional activities.	Public benchmark datasets; USC-HAD included indoor and outdoor sessions. Other setting details N/A.	Benchmark HAR classification of daily and transitional activities. KU-HAR includes stand, sit, walking, running, stair up/down, stand-sit, lay-stand, and other activities; UCI-HAR includes walking, walking upstairs/downstairs, sitting, standing, and lying; USC-HAD includes walking, running, jumping, sitting, standing, sleeping, and elevator transitions.	Sit-to-stand; Stand-to-sit,
Al-qaness et al. (2024) [52]	Secondary dataset analysis.	HAR/fall classification including transition-related classes.	Deep learning: parallel CNN–Transformer architecture.	Participants/recordings from three public fall and HAR datasets: SisFall, UniMiB-SHAR, and MobiAct.	SisFall: 38 participants (15 elderly, 23 young adults); UniMiB-SHAR: 30 human subjects and 11,771 samples; MobiAct: 57 men and women. Evaluation sample counts varied by dataset/table.	No clinical patient group reported; public datasets of activities of daily living and simulated/recorded falls, including elderly and young adults in SisFall.	Public-dataset protocols; specific environmental setting for each dataset was not reported in detail in this paper.	Public HAR/fall protocols including walking, jogging/running, stairs, sitting/lying, standing, jumping, car-step in/out, stand-to-sit, sit-to-stand, and multiple fall scenarios.	Sit-to-stand; Stand-to-sit; Rolling/turning in bed.
Amici et al. (2024) [10]	Cross-sectional comparative study.	PT quantification; known-groups discrimination.	Rule-/threshold-based event detection + signal-derived feature computation.	Healthy volunteers and pathological subjects recruited within the first week of rehabilitation with a prescription for rehabilitation of postural transitions.	N = 33; healthy subjects n = 20; pathological subjects n = 13; final dataset composed of 495 acquisitions.	Healthy subjects and heterogeneous pathological rehabilitation sample: 8 post-stroke survivors, 2 subjects with Guillain–Barrè–Strohl syndrome, 1 with multiple sclerosis, 1 with paraparesis, and 1 outcome of laminectomy.	Clinical/rehabilitation context; standard physiotherapy bed used for the acquisition protocol.	Custom protocol with five postural transitions: SiSu, SuSi, SiSt, StSi, and Roll.	Sit-to-stand; stand-to-sit; supine-/lying-to-sit; sit-to-supine/sitting-to-lying; rolling/turning in bed.
Atrsaei et al. (2020) [11]	Experimental laboratory study.	Detection; segmentation; PT-type classification; quantification.	Wavelet-/model-based rule algorithm: CWT peak detection + sigmoid fitting.	Dataset A: young healthy adults; Dataset B: healthy younger adults, healthy older adults, idiopathic Parkinson’s disease, multiple sclerosis, stroke.	Total N = 57; Dataset A n = 15; Dataset B n = 42.	Healthy younger adults; healthy older adults; idiopathic Parkinson’s disease (IPS); multiple sclerosis (MS); stroke.	Laboratory and simulated daily-life/home-like setting.	Dataset A test 1: 10 min daily tasks; Dataset A test 2: 5×STS on chair without armrest; Dataset B: simulated home-setting daily-living tasks.	Sit-to-stand; stand-to-sit.
Atrsaei et al. (2021) [29]	Cross-sectional comparative study.	PT quantification; clinical discrimination; daily-life detection/counting.	Rule-based published algorithms + conventional ML decision-tree classification.	Patients with Parkinson’s disease.	N = 26; PD-FOF+ n = 9; PD-FOF− n = 17.	Parkinson’s disease; PD with Fear of Falling (PD-FOF+, FES-I score >19) and PD without Fear of Falling (PD-FOF−).	Laboratory/clinical assessment and daily activity/home environment.	Lab: 7 m Timed Up and Go (TUG) test at convenient speed; 5×STS test once at preferred speed and once as fast as possible. Daily activity: 14 days of monitoring.	Sit-to-stand; stand-to-sit.
Bagalà et al. (2013) [2]	Cross-sectional comparative study with test–retest component.	Event detection/segmentation; quantification; group discrimination.	Rule-based event detection + extended Kalman filter + ROC/statistical analysis.	Young healthy hospital employees and older patients from a geriatric rehabilitation clinic.	Total N = 25; young healthy subjects n = 10; older geriatric rehabilitation patients n = 15.	Young healthy subjects; older geriatric rehabilitation patients. Older patients included participants after hip fracture, other fractures, or decompensation after acute internal illness; inclusion criteria also allowed disorders affecting body symmetry or making transfers difficult.	Experimental clinical/rehabilitation setting using a conventional nursing bed in a geriatric rehabilitation/hospital context.	Lying-to-sit-to-stand-to-walk transfer from a conventional nursing bed.	Sit-to-stand; supine-/lying-to-sit
Braun et al. (2022) [30]	Prospective observational cohort with repeated measures.	PT quantification; clinical association.	No PT-specific algorithm; quantitative feature extraction + regression analysis.	Older geriatric inpatients with heart failure and a heart-healthy comparison sub-sample without known structural heart disease and NT-proBNP <125 pg/mL.	Total N = 61; heart failure n = 49; no structural heart disease n = 12.	Heart failure classified by NYHA class: no HF n = 12, NYHA I n = 0, NYHA II n = 26, NYHA III n = 13, NYHA IV n = 10.	Clinical inpatient setting; supervised hospital ward on level ground.	SPPB and TUG performed at admission and discharge; SPPB included standing balance, 4 m walk, and 5-times chair rise; TUG included chair rise, 3 m walk, turn, walk back, and return to chair.	Sit-to-stand
Buchman et al. (2020) [31]	Prospective longitudinal cohort study.	PT quantification; prognostic association.	Previously validated rule-based formulas + longitudinal association models.	Community-dwelling older adults from the Rush Memory and Aging Project and the Minority Aging Research Study.	Total N = 1249; Rush Memory and Aging Project n = 766; Minority Aging Research Study n = 483.	Older adults; no single clinical condition group. Outcomes included mortality, incident ADL disability, mobility disability, mild cognitive impairment, and Alzheimer’s disease dementia.	Home/residence setting, with structured supervised testing.	Structured mobility testing at participants’ residence: 32-ft walk, Timed Up and Go, and 20 s standing posture.	Sit-to-stand; stand-to-sit.
Caronni et al. (2018) [32]	Retrospective cross-sectional study.	TUG segmentation; quantification; criterion validity.	Manual segmentation + partial least squares regression.	Neurological patients attending a rehabilitation clinic, with a wide range of balance and gait impairments.	N = 122.	Stroke n = 45; lower-limb polyneuropathy n = 30; Parkinson’s disease and parkinsonisms n = 26; other diagnoses n = 21, including myelopathy n = 9, motor neuron disease n = 6, traumatic brain injury n = 3 and surgery because of brain tumor n = 3.	Clinical rehabilitation setting; corridor close to the rehabilitation gym, 2 × 6 m.	Conventional 3 m Timed Up and Go Test performed with an IMU; Mini-BESTest and FIM also administered.	Sit-to-stand; stand-to-sit.
Chen et al. (2024) [41]	Experimental laboratory study.	STS phase segmentation and classification.	Hybrid thresholding + deep learning (CNN–BiLSTM–Attention).	Healthy young adults without reported musculoskeletal diseases or severe neurological injuries.	N = 15 subjects; 12 male/3 female. Customized STS-PD dataset: 27,276 windowed samples split into 19,101 training and 8175 testing samples.	No clinical patient group; healthy subjects recruited to build and validate the sit-to-stand phase-identification dataset. Application context: Wearable exoskeleton assistance for patients with lower-limb motor dysfunction.	Laboratory experimental setup with two force plates, two Xsens MTw IMUs, signal amplifiers, Awinda Station receiver, and workstation.	Sit-to-stand transitions from an armless/backless seat.	Sit-to-stand.
Coulthard et al. (2015) [59]	Repeated-measures experimental study.	TUG subtask quantification; dual-task comparison.	Commercial software-based segmentation + statistical analysis.	Healthy English-speaking young adults.	N = 20; 10 male and 10 female.	Healthy young adults; participants were excluded if they had a lower-limb injury or lower-limb surgery within the previous 6 months, neurological disease, or recent concussion.	Laboratory setting.	Instrumented TUG using a 7 m return point, performed in control, mental tracking, and verbal fluency conditions.	Sit-to-stand; Stand-to-sit.
Ganea et al. (2011) [33]	Comparative observational study with pre–post component.	Detection/segmentation; quantification; group discrimination.	Rule-based trunk-tilt segmentation + ROC/statistical analysis.	Frail elderly subjects admitted to a post-acute rehabilitation facility and healthy community-dwelling elderly persons.	Total N = 106; Frail Elderly n = 79; Healthy Elderly n = 27.	Frail Elderly subjects fulfilling Fried’s frailty criteria; Healthy Elderly community-dwelling subjects.	Controlled clinical/rehabilitation and community assessment setting.	Measurement protocol with one sit-to-stand followed by one stand-to-sit transition using a standard chair with armrests; frail subjects were also assessed before and after a three-week rehabilitation program.	Sit-to-stand; Stand-to-sit.
Kirchberger et al. (2025) [53]	Monocentric feasibility study.	Activity classification; sensor-location comparison.	Conventional machine learning: Random Forest.	Adult acute-care hospital patients at University Hospital Basel who were able to walk before admission and cognitively able to follow instructions.	N = 40 recruited; 86 measurements; 56 usable recordings from 29 patients for model training/evaluation; final mirrored dataset included 10.9 h of annotated activity data per sensor location.	Acute-care hospital patients, mainly vascular surgery (75%), trauma orthopedics (17.5%), and other departments (7.5%); 40% used a walking aid.	Acute-care hospital environment at University Hospital Basel.	Standardized physical assessment: sensor calibration, supine lying, sitting, 10 sit-to-stand transitions, standing, walking 10 or 40 m, and stairs if feasible.	Sit-to-stand.
Kuo et al. (2023) [42]	Experimental laboratory study.	Posture/transition classification; transition-time prediction.	Deep learning: LSTM/FNN classification.	Healthy volunteers recruited as a convenience sample; none had current or previous neurological or orthopedic pathology of the right leg.	N = 30 healthy subjects.	Healthy young/adult participants; no clinical condition or patient group.	Controlled experimental/laboratory setting.	Series of stand–walk–stand–sit cycles lasting 5–6 min.	Sit-to-stand; stand-to-sit.
Lin et al. (2023) [34]	Cross-sectional diagnostic classification study.	TUG segmentation/quantification; diagnostic discrimination.	Rule-based TUG segmentation + conventional ML ensemble (SVM/RF/logistic regression).	Patients with early-stage Parkinson’s disease and patients with Essential Tremor were recruited at Ruijin Hospital, Shanghai Jiao Tong University School of Medicine.	Total N = 164; early-stage PD n = 84; Essential Tremor n = 80. Training dataset: PD n = 64, ET n = 67; independent test dataset: PD n = 20, ET n = 13.	Early-stage Parkinson’s disease, Hoehn and Yahr stage 1–1.5; Essential Tremor patients with limb tremor symptoms.	Clinical/hospital setting at Ruijin Hospital, Shanghai Jiao Tong University School of Medicine.	Instrumented Timed Up and Go Test: stand up from a chair, walk 5 m at a comfortable speed, turn 180°, walk back, turn 180° in front of the chair, and sit down.	Sit-to-stand; stand-to-sit.
Marin et al. (2022) [35]	Cross-sectional observational study.	STS quantification/scoring; clinical association.	Signal-feature modeling (PCA) + unsupervised k-means + regression.	Parkinson’s disease patients and asymptomatic young and senior adults.	Total unique participants: N = 70; Parkinson’s disease n = 20; asymptomatic participants n = 50 (young adults n = 33, senior adults n = 17). Ten PD measurements were in the OFF state, 17 in the ON state, and seven PD patients were measured in both ON and OFF states.	Parkinson’s disease; asymptomatic young adults; asymptomatic senior adults. PD patients included medication ON and/or OFF assessments.	Clinical/research assessment setting; data were collected as part of a larger project at Kiel University/University Hospital Schleswig-Holstein.	Five sit-to-stand/chair-rise test from a standard seat height.	Sit-to-stand.
Martinez-Hernandez et al. (2019) [43]	Experimental laboratory study.	State/phase detection and classification.	Probabilistic Bayesian sequential model.	Healthy adults from the School of Mechanical Engineering, University of Leeds; participants were free from gait abnormalities and neurological pathologies.	N = 12.	Healthy participants; no clinical/pathological group.	Laboratory data collection with offline and real-time validation; additional simulated robotic-leg environment for controller interaction.	Participants performed sit-to-stand and stand-to-sit activities; a sequential activity validation also included walking, sit down, stand up, ramp ascent, walking, ramp descent, walking, sit down, stand up, and walking.	Sit-to-stand; stand-to-sit.
Mazumder et al. (2016) [44]	Experimental laboratory study.	Ambulatory activity classification.	Conventional machine learning: dendrogram-based SVM.	Healthy adult volunteers.	n = 5.	Healthy participants; no clinical condition reported.	Laboratory/offline algorithm-testing setting, including treadmill and stairs; application intended for lower-limb exoskeleton control.	Sit-to-stand and stand-to-sit protocol; level walking, slope walking, fast walking on treadmill; stair ascent and stair descent; static sit and stand states.	Sit-to-stand; stand-to-sit.
Najafi et al. (2003) [45]	Multi-setting methodological validation study.	PT/activity detection and classification; free-living monitoring.	Wavelet-/kinematic rule-based classification (DWT + kinematic model).	Elderly persons older than 65 years: community-dwelling elderly subjects, hospitalized elderly rehabilitation patients, and elderly subjects monitored during daily activity.	Study 1: n = 11; Study 2: n = 24; Study 3: n = 9.	Elderly participants; Study 2 included hospitalized elderly persons in a rehabilitation unit. Specific diagnoses were N/A.	Gait laboratory, clinical/rehabilitation unit, and free-living/daily-activity environment.	Study 1: Six laboratory tests including lying down, walking, sit-to-stand, and stand-to-sit with bed and different chairs with/without armrests. Study 2: Lying-position changes and transfers in/out of bed. Study 3: 45–60 min of daily physical activity; a 12 h monitoring example was also shown.	Sit-to-stand; stand-to-sit; supine/lying-to-sit; sit-to-supine/sitting-to-lying; rolling/turning in bed.
Netuková et al. (2021) [36]	Cross-sectional observational study.	Signal characterization and PT quantification.	Threshold-based detection + continuous-curve statistical analysis.	Older adult volunteers and de novo Parkinson disease patients.	Total n = 60; control group n = 29; Parkinson disease group n = 31.	Control group: Older adult volunteers free from neurological or psychiatric disorder; Parkinson disease group: De novo PD patients.	N/A.	Instrumented extended 10 m Timed Up and Go Test.	Sit-to-stand.
Nishizawa et al. (2024) [37]	Cross-sectional observational study.	TUG subphase quantification; clinical association/discrimination.	No PT-specific algorithm; statistical classification (logistic regression/ROC).	Middle-aged adults under 65 years of age recruited during visits to the hospital for a health checkup.	Total N = 97; non-LS n = 57; LS n = 40.	Locomotor syndrome versus non-locomotor syndrome. LS classification: 39 participants were LS-stage-1 and 1 participant was LS-stage-2. Participants had no neurological disease, neuromuscular disease, or cognitive decline.	Participants were recruited during hospital health checkups; the exact measurement environment for the TUG was N/A.	3 m Timed Up and Go Test at comfortable and fast speeds; LS tests were used to determine locomotive-syndrome stage.	Sit-to-stand; stand-to-sit.
Parr et al. (2025) [38]	Cross-sectional secondary analysis.	STS quantification; group comparison.	Threshold-/slope-based segmentation + descriptive/statistical analysis.	Persons with unilateral transtibial lower-limb amputation recruited from amputation-related events, military treatment facilities, and a prosthetics clinic.	N = 58; no low back pain n = 28; low back pain n = 30.	Unilateral transtibial amputation; comparison between participants with low back pain and without low back pain.	Study testing with participants recruited from amputation-related events, military treatment facilities, and a local prosthetics clinic; specific laboratory/clinical room setting was N/A.	Five sit-to-stand and subsequent stand-to-sit cycles/tasks.	Sit-to-stand; stand-to-sit.
Parvaneh et al. (2017) [3]	Cross-sectional observational study.	Free-living PT quantification; frailty discrimination.	Rule-based posture-sequence algorithm + logistic regression.	Cognitively intact community-dwelling older adults aged 65 years and older, without gait or mobility disorders and able to walk 20 m with or without an assistive device.	N = 120 recruited; n = 119 analyzed after exclusion of one frail subject based on walking percentage outlier. Non-frail n = 43; pre-frail/frail n = 76.	Non-frail versus pre-frail/frail groups classified using Fried’s frailty criteria; pre-frail and frail participants were combined.	Unsupervised/natural daily-living environment, including community/home or assisted-living settings.	Spontaneous daily physical activity monitoring over 48 h, with the first 24 h used for the study.	Sit-to-stand; stand-to-sit
Pham et al. (2018) [46]	Experimental validation study.	PT detection and quantification; algorithm validation.	Rule-based zero-crossing + DWT denoising/sensor fusion/kinematic estimation.	Parkinson’s disease patients and older adults. Participants were examined by a movement disorder specialist; included if they had no orthopedic problems and were able to walk without aids; excluded for deep brain stimulation or MMSE <24.	Training group: n = 5 (4 PD patients, including 2 with dyskinesia, and 1 older adult). Test group: n = 32 (21 PD patients, including 16 without dyskinesia and 5 with dyskinesia, and 11 older adults).	PD patients with and without dyskinesia; older adults. Clinical descriptors reported as MDS-UPDRS III, Hoehn & Yahr, and levodopa equivalent dose for PD/OA groups.	Home-like/semi-naturalistic environment.	Daily-life activities in home-like environment.	Sit-to-stand; stand-to-sit.
Regterschot et al. (2014) [58]	Test–retest repeated-measures study.	Reliability of quantitative STS measures.	Rule-based event definition + signal integration/power computation.	Healthy young female adults and healthy older adults. Older adults were included if aged ≥70 years, able to walk 10 m with a wheeled walker/cane allowed, and able to stand up from a chair; major cardiovascular/respiratory, neurological, severe comorbidity, cognitive, recent orthopedic surgery, severe visual problems, and recent stroke criteria were used as exclusions.	Total N = 41; young adults n = 10; older adults n = 31.	Healthy young female adults; healthy older adults. Thirteen older adults reported at least one fall in the previous year.	N/A for exact testing environment. Participants were assessed in two test–retest sessions; older adults were recruited from a residential-care home, sheltered houses, and a health care center.	Normal and fast sit-to-stand transfers; older adults also performed FTSST and TUGT.	Sit-to-stand.
Salarian et al. (2010) [55]	Cross-sectional comparative study with test–retest component.	TUG segmentation/quantification; known-groups validity and reliability.	Rule-based/commercial inertial segmentation + model fitting/statistical analysis.	Twelve subjects with idiopathic Parkinson’s disease in early-to-moderate stages and twelve age-matched healthy control subjects.	N = 24 for group comparison; n = 12 PD and n = 12 controls. Test–retest reliability: n = 18, with nine subjects from each group repeating the protocol.	Early-to-moderate idiopathic Parkinson’s disease versus age-matched healthy controls; PD participants were unmedicated and had H&Y scores 1–2.5 and UPDRS motor score 20.0 ± 9.4.	Clinical/laboratory assessment setting at Oregon Health & Science University; exact room/lab details: N/A.	Timed Up and Go and instrumented TUG. Standard TUG used a 3 m walkway; iTUG used a 7 m walkway.	Sit-to-stand; stand-to-sit
Suglia et al. (2024) [54]	Experimental laboratory study.	Continuous HAR classification including PT-related classes.	Deep learning: multi-branch 1D CNN.	Healthy adults recruited from the staff of IRCCS Maugeri, Bari, Italy.	N = 18 healthy subjects. Training dataset: 10 subjects (4 males, 6 females), 300 separate-task acquisitions. Test dataset: 8 different subjects (4 males), 40 uninterrupted-circuit acquisitions.	No clinical condition/group reported; all subjects were healthy, right-handed, and without motor or cognitive pathologies.	MARLab—Movement Analysis and Robotic Lab—IRCCS Maugeri, Bari, Italy.	Separate training protocol: walking + turning, sit-to-stand, and lying-down tasks. Continuous test protocol: sitting start, stand up, walk 7 m, turn 180°, walk back 7 m to a couch, turn, lie down, and keep lying for about 5 s.	Sit-to-stand.
Van Lummel et al. (2012) [47]	Cross-sectional criterion-validation study.	Seat-off/seat-on event detection; criterion validation.	Feature-/model-based event prediction + leave-one-out cross-validation.	Older adults recruited from a residential-care home and young adults recruited among college students. Young adults had no history of neuromuscular or musculoskeletal disorders.	Recruited n = 30 (15 older adults and 15 young adults). Two older adults were unable to stand up and were excluded; 15 young adults and 13 older adults performed two sets of five trials. Overall, 253 trials were analyzed successfully.	Older adults living in a residential-care home and healthy young adults.	N/A for exact laboratory/clinical setting; older adults were recruited from a residential-care home and young adults from college students.	Repeated sit-to-stand task: Two sets of five sit-to-stand-to-sit cycles at self-selected speed on a standard chair without armrests, height 42 cm.	Sit-to-stand; stand-to-sit.
Wairagkar et al. (2022) [48]	Experimental validation and application study.	PT segmentation/classification; kinematic estimation.	Biomechanical/model-based estimation + extended Kalman filter + unsupervised k-means classification.	Younger healthy adults, older healthy adults, and people with Parkinson’s disease.	Total N = 34; younger healthy adults n = 10; older healthy adults n = 12; people with Parkinson’s disease n = 12.	Younger healthy adults; older healthy adults; people with Parkinson’s disease. In the Parkinson’s group, eight participants had Hoehn and Yahr score 3, one had 2.5, one had 2, and two had 1.5.	Younger healthy adults were tested at the University of Reading. Older healthy adults and people with Parkinson’s were tested in the University of Southampton study; data from 4 older healthy and 10 Parkinson’s participants were collected at home, and data from the remaining participants in those groups were collected in the laboratory.	Sit-to-stand and stand-to-sit transition protocol.	Sit-to-stand; stand-to-sit
Wang et al. (2024) [39]	Cross-sectional observational study.	TUG subtask quantification; fall-risk discrimination.	Rule-based TUG segmentation + statistical/logistic classification.	Community-dwelling older adults aged 65 years and above recruited from four community settings in Hong Kong.	N = 41 completed all tests; 36 women and 5 men; 15 high-fall-risk and 26 low-fall-risk participants based on Berg Balance Scale scores.	Community-dwelling older adults classified as high-fall-risk or low-fall-risk using BBS cut-off: low risk if BBS >50, high risk if BBS ≤ 50.	Four community settings in Hong Kong; data collection occurred between December 2019 and January 2020.	3 m Timed Up and Go Test; BBS was used for fall-risk grouping.	Sit-to-stand; stand-to-sit.
Wang et al. (2025) [49]	Experimental laboratory study.	STS phase classification; joint-angle prediction.	Deep learning: Transformer dual-task model.	Healthy subjects and stroke patients performing sit-to-stand movements.	N = 14; 7 healthy subjects and 7 stroke patients. Each subject performed the sit-to-stand task approximately 10 times.	Healthy subjects and stroke patients; the patient group included unilateral lower-limb motor dysfunction with a paretic side, and the FMA-LE score was reported.	N/A.	Sit-to-stand task from a height-adjustable chair.	Sit-to-stand
Witchel et al. (2018) [57]	Cross-sectional comparative study with within-session repeatability.	PT quantification; clinical discrimination.	Rule-/feature-based segmentation + statistical discrimination.	Ambulatory persons with multiple sclerosis and middle-aged healthy volunteers.	Total N = 40; 17 persons with multiple sclerosis and 23 healthy volunteers.	Persons with multiple sclerosis (PwMS; Hauser Ambulation Index ≤2, no walking aids for short distances) and healthy volunteers.	PwMS were recruited from a local community MS center and healthy volunteers from the university community; testing setting otherwise N/A.	Timed Up and Go Test with 3 m walkway; the task was performed twice.	Sit-to-stand; stand-to-sit
Zakaria et al. (2015) [40]	Cross-sectional experimental study.	TUG segmentation/quantification; fall-risk classification.	Rule-based TUG phase segmentation + conventional ML k-NN classification.	Elderly people from Fujimoto Hayasuzu Hospital, Japan.	Total N = 38; 20 male and 18 female. Low fall risk n = 27 and high fall risk n = 11 according to the k-NN/test-subject description; the paper also reports grouping by a 13.5 s TUG threshold.	Low-fall-risk and high-fall-risk older adults, categorized using the 13.5 s TUG completion-time threshold.	Hospital/clinical setting at Fujimoto Hayasuzu Hospital, Japan.	Timed Up and Go Test: sit on an armless chair with back, stand up, walk 3 m to a marked post, turn, walk back 3 m, turn, and sit.	Sit-to-stand; stand-to-sit.
Zijlstra et al. (2010) [50]	Experimental criterion-validation study.	STS quantification; criterion validation.	Biomechanical/signal-integration model + force-plate criterion comparison.	Healthy young adults and healthy older adults.	Total N = 17; 5 healthy young subjects and 12 healthy older subjects.	Healthy young adults and healthy older adults; older participants had no fall history and were able to stand up from a chair without help.	Laboratory setting with force plates and Optotrak motion analysis system.	Sit-to-stand from a standard-height chair (0.46 m).	Sit-to-stand.

Abbreviations: ADL: Activity of Daily Living; BEST: Balance Evaluation System Test; ET: Essential Tremor; FES-I: Falls Efficacy Scale-International; FIM: Functional Independence Measure; FOF: Fear of Falling; FTSST: Five Time Sit-to-Stand Test; HAR: Human Activity Recognition; IMU: Inertial Measurement Unit; ITUG: Instrumented Timed Up and Go; LS: Locomotive Syndrome; LSSW: lie to sit to stand to ealk; m: meters; MDS-UPDRS: Movement Disorder Society-sponsored revision of the Unified Parkinson’s Disease Rating Scale; NYHA: New York Heart Association; N/A: not available or not reported; PD: Parkinson’s Disease; PT: postural transition; SPPB: Short Physical Performance Battery; SiSt: sit to stand; SiSu: sit to supine; STS: sit-to-stand; StSi: stand to sit; SuSi: supine to sit; TUG: Timed Up and Go; TUGT: Timed Up and Go Test; and 10MWT: 10 m walk test. Note: For fully extracted details, see Supplementary Material S3—Table S3.

**Table 3:** Methodology appraisal of the included studies.

**Study**	**Study Design/** **Purpose**	**Appraisal Tool**	**Main Methodological Concerns**	**Overall Judgment**
Abdollahi et al., 2024 [56]	Cross-sectional clinical assessment	JBI + COSMIN-i	No PT-specific segmentation or external validation; limited reliability/agreement; modest sample and confounder control	Moderate-to-high
Abraha et al., 2026 [51]	Secondary HAR/algorithm validation	COSMIN-i	Benchmark datasets; window-level classification; limited participant-independent/external validation; no PT event-timing validation	Moderate-to-high
Al-Qaness et al., 2024 [52]	Secondary deep-learning HAR validation	COSMIN-i	No PT-specific segmentation; no external validation; class imbalance; limited transition-class samples	Moderate-to-high
Amici et al., 2024 [10]	Cross-sectional PT quantification/validity	JBI + COSMIN-i	Small heterogeneous sample; no external biomechanical reference; no reliability/agreement analysis	Moderate
Atrsaei et al., 2020 [11]	Algorithm-validation study	JBI + COSMIN-i	Sensor-location effects; limited independent validation; incomplete robustness evidence	Moderate
Atrsaei et al., 2021 [29]	Cross-sectional clinical study	JBI	Small sample; limited confounder adjustment; possible classification overfitting	Moderate
Bagalà et al., 2013 [2]	Methodological/test–retest study	COSMIN-i	Small sample; limited reliability reporting; no SEM/MDC or ICC confidence intervals	Moderate
Braun et al., 2022 [30]	Cross-sectional association study	JBI	Small sample; missing data; exploratory analyses; limited confounder adjustment	Moderate
Caronni et al., 2018 [32]	Criterion-validity study	JBI + COSMIN-i	Heterogeneous neurological sample; manual segmentation; no reliability/measurement-error analysis	Moderate
Buchman et al., 2020 [31]	Longitudinal/prognostic cohort	QUIPS	PT metrics associated with outcomes, but PT detection/segmentation not validated	Moderate
Chen et al., 2024 [41]	Algorithm-validation study	COSMIN-i	Small healthy sample; random split/augmentation; potential overfitting; no patient or reliability validation	Moderate
Coulthard et al., 2015 [59]	Repeated-measures clinical-test study	JBI + COSMIN-i	Healthy young sample; limited agreement/reliability evidence	Moderate
Ganea et al., 2011 [33]	Comparative observational study	JBI + COSMIN-i	Non-age-matched groups; no confounder adjustment; no reliability or biomechanical reference	Moderate
Kirchberger et al., 2025 [53]	Feasibility/algorithm-development study	JBI + COSMIN-i	Activity-class rather than PT segmentation; class imbalance/data loss; no free-living or external validation	Moderate
Kuo et al., 2023 [42]	Algorithm-validation study	COSMIN-i	Healthy convenience sample; manual labeling; limited reference comparison; no reliability/agreement	Moderate
Lin et al., 2023 [34]	Diagnostic classification study	JBI	High-dimensional feature selection; overfitting risk; small test set; no external validation	Moderate-to-high
Marin et al., 2022 [35]	Sensor-based validation study	COSMIN-i	Small PD sample; limited discrimination; no reliability or external biomechanical validation	Moderate
Martinez-Hernandez et al., 2019 [43]	Algorithm-validation study	COSMIN-i	Small healthy sample; limited reference labeling; no external biomechanical or reliability validation	Moderate
Mazumder et al., 2016 [44]	Activity-classification validation	COSMIN-i	Very small healthy sample; offline validation; no independent/external validation	Moderate
Najafi et al., 2003 [45]	Ambulatory algorithm-validation study	COSMIN-i	Small elderly samples; observer-based references; limited reliability/agreement statistics	Some concerns
Neťuková et al., 2021 [36]	IMU measurement/validity study	COSMIN-i	No external biomechanical reference; limited repeatability; no measurement-error/agreement analysis	Moderate
Nishizawa et al., 2024 [37]	Cross-sectional association study	JBI	Limited confounder adjustment; sex imbalance; no reliability or longitudinal validation	Moderate
Parr et al., 2025 [38]	Cross-sectional PT quantification study	JBI + COSMIN-i	No external metric/event validation; non-standardized chair height; no reliability/measurement error	Moderate
Parvaneh et al., 2017 [3]	Cross-sectional frailty study	JBI	Small frail subgroup; residual confounding; no independent validation of transition counting	Moderate
Pham et al., 2018 [46]	PT algorithm-validation study	COSMIN-i	Small training dataset; limited precision of video reference; variable agreement; no reliability analysis	Moderate
Regterschot et al., 2014 [58]	Test–retest reliability study	COSMIN-i	No external criterion validation; no MDC/Bland–Altman analysis; limited generalizability	Some concerns
Salarian et al., 2010 [55]	Reliability/known-groups validity study	COSMIN-i	Small sample; short retest interval; no SEM/MDC; incomplete external validation	Moderate
Suglia et al., 2024 [54]	Secondary deep-learning HAR validation	COSMIN-i	STS treated as activity class; no PT event validation; no external reference or clinical validation	Moderate-to-high
Van Lummel et al., 2012 [47]	PT event-detection validation	COSMIN-i	Small sample; semi-automatic analysis; trial exclusions; no clinical validation	Some concerns
Wairagkar et al., 2022 [48]	PT modeling/classification study	COSMIN-i	Small samples; limited external validation; model assumptions; no reliability/event-timing validation	Moderate
Wang et al., 2024 [39]	Cross-sectional fall-risk study	JBI	Small sex-imbalanced sample; surrogate fall-risk definition; multiple testing; no external/reliability validation	Moderate-to-high
Wang et al., 2025 [49]	Algorithm-validation study	COSMIN-i	Small sample; intra-subject/window-level validation; limited cross-subject and real-world validation	Moderate-to-high
Witchel et al., 2018 [57]	PT quantification study	COSMIN-i	Small MS sample; many exploratory features; limited repeatability; no external validation/agreement	Moderate
Zakaria et al., 2015 [40]	Cross-sectional TUG study	JBI	Potential circularity in fall-risk definition; small sample; no confounder adjustment or external validation	Moderate-to-high
Zijlstra et al., 2010 [50]	Criterion-validation study	COSMIN-i	Small healthy sample; laboratory setting; no test–retest reliability or agreement analysis	Some concerns

Abbreviations: BBS: Berg Balance Scale; COSMIN: COnsensus-based Standards for the selection of health Measurement INstruments; JBI: Joanna Briggs Institution; MDC: Minimal Detectable Change; PT: postural transition; SEM: Standard Error of Measurement; QUIPS: Quality In Prognosis Studies tool; TUG: Timed Up and Go Test; STS: Sit to Stand; and StSi: Stand to Sit. Note: COSMIN-i, COSMIN-informed appraisal; JBI, Joanna Briggs Institute critical appraisal tool; and QUIPS, Quality In Prognosis Studies. Where a study addressed both observational and measurement-property objectives, JBI and COSMIN-informed domains were combined. No numerical scoring or weighting was applied.

**Table 4:** Postural transitions assessed in the included studies.

**Postural Transitions Assessed**	**N° of Studies**	**Studies**
Sit-to-stand	35	All included studies
Stand-to-sit	25	Abdollahi et al., 2024 [56]; Abraha et al., 2026 [51]; Al-Qaness et al., 2024 [52]; Amici et al., 2024 [10]; Atrsaei et al., 2020 [11]; Atrsaei et al., 2021 [29]; Buchman et al., 2020 [31]; Caronni et al., 2018 [32]; Coulthard et al., 2015 [59]; Ganea et al., 2011 [33]; Kuo et al., 2023 [42]; Lin et al., 2023 [34]; Martinez-Hernandez et al., 2019 [43]; Mazumder et al., 2016 [44]; Najafi et al., 2003 [45]; Nishizawa et al., 2024 [37]; Parr et al., 2025 [38]; Parvaneh et al., 2017 [3]; Pham et al., 2018 [46]; Salarian et al., 2010 [55]; Van Lummel et al., 2012 [47]; Wairagkar et al., 2022 [48]; Wang et al., 2024 [39]; Witchel et al., 2018 [57]; Zakaria et al., 2015 [40]
Supine-/lying-to-sit	3	Amici et al., 2024 [10]; Bagala et al., 2013 [2]; Najafi et al., 2003 [45]
Sit-to-supine/sitting-to-lying	2	Amici et al., 2024 [10]; Najafi et al., 2003 [45]
Rolling/turning in bed	3	Al-Qaness et al., 2024 [52]; Amici et al., 2024 [10]; Najafi et al., 2003 [45]

**Table 5:** Summary of IMU setup and acquisition protocols across the included studies.

	**Category**	**No. of Studies**	**Studies**
Sensor configuration	Single IMU/sensor	17	Amici et al., 2024 [10]; Atrsaei et al., 2020 [11]; Atrsaei et al., 2021 [29]; Bagala et al., 2013 [2]; Buchman et al., 2020 [31]; Caronni et al., 2018 [32]; Ganea et al., 2011 [33]; Marin et al., 2022 [35]; Martinez-Hernandez et al., 2019 [43]; Najafi et al., 2003 [45]; Netukova et al., 2021 [36]; Parvaneh et al., 2017 [3]; Pham et al., 2018 [46]; Regterschot et al., 2014 [58]; Van Lummel et al., 2012 [47]; Wang et al., 2024 [39]; Zakaria et al., 2015 [40]
	Multi-IMU setup	18	Abdollahi et al., 2024 [56]; Atrsaei et al., 2020 [11]; Atrsaei et al., 2021 [29]; Braun et al., 2022 [30]; Chen et al., 2024 [41]; Coulthard et al., 2015 [59]; Kirchberger et al., 2025 [53]; Kuo et al., 2023 [42]; Lin et al., 2023 [34]; Mazumder et al., 2016 [44]; Nishizawa et al., 2024 [37]; Parr et al., 2025 [38]; Salarian et al., 2010 [55]; Suglia et al., 2024 [54]; Wairagkar et al., 2022 [48]; Wang et al., 2025 [49]; Witchel et al., 2018 [57]; Zijlstra et al., 2010 [50]
	Smartphone or benchmark inertial dataset	2	Abraha et al., 2026 [51]; Al-Qaness et al., 2024 [52]
Body placement	Trunk-/pelvis-/waist-based placement	31	Abdollahi et al., 2024 [56]; Amici et al., 2024 [10]; Atrsaei et al., 2020 [11]; Atrsaei et al., 2021 [29]; Bagala et al., 2013 [2]; Braun et al., 2022 [30]; Buchman et al., 2020 [31]; Caronni et al., 2018 [32]; Chen et al., 2024 [41]; Coulthard et al., 2015 [59]; Ganea et al., 2011 [33]; Kuo et al., 2023 [42]; Lin et al., 2023 [34]; Marin et al., 2022 [35]; Mazumder et al., 2016 [44]; Najafi et al., 2003 [45]; Netukova et al., 2021 [36]; Nishizawa et al., 2024 [37]; Parr et al., 2025 [38]; Parvaneh et al., 2017 [3]; Pham et al., 2018 [46]; Regterschot et al., 2014 [58]; Salarian et al., 2010 [55]; Suglia et al., 2024 [54]; Van Lummel et al., 2012 [47]; Wairagkar et al., 2022 [48]; Wang et al., 2024 [39]; Wang et al., 2025 [49]; Witchel et al., 2018 [57]; Zakaria et al., 2015 [40]; Zijlstra et al., 2010 [50]
	Lower-limb placement	15	Abdollahi et al., 2024 [56]; Atrsaei et al., 2021 [29]; Braun et al., 2022 [30]; Chen et al., 2024 [41]; Coulthard et al., 2015 [59]; Kirchberger et al., 2025 [53]; Kuo et al., 2023 [42]; Lin et al., 2023 [34]; Martinez-Hernandez et al., 2019 [43]; Mazumder et al., 2016 [44]; Parr et al., 2025 [38]; Salarian et al., 2010 [55]; Wairagkar et al., 2022 [48]; Wang et al., 2025 [49]; Witchel et al., 2018 [57]
	Trunk + lower limb/multi-segment placement	13	Abdollahi et al., 2024 [56]; Atrsaei et al., 2021 [29]; Braun et al., 2022 [30]; Chen et al., 2024 [41]; Coulthard et al., 2015 [59]; Kuo et al., 2023 [42]; Lin et al., 2023 [34]; Mazumder et al., 2016 [44]; Parr et al., 2025 [38]; Salarian et al., 2010 [55]; Wairagkar et al., 2022 [48]; Wang et al., 2025 [49]; Witchel et al., 2018 [57]
	Pocket/waist smartphone or dataset-defined placement	2	Abraha et al., 2026 [51]; Al-Qaness et al., 2024 [52]
Sampling frequency	<50 Hz	4	Ganea et al., 2011 [33]; Kuo et al., 2023 [42]; Najafi et al., 2003 [45]; Wang et al., 2024 [39]
	50–100 Hz	19	Abdollahi et al., 2024 [56]; Abraha et al., 2026 [51]; Al-Qaness et al., 2024 [52]; Bagala et al., 2013 [2]; Buchman et al., 2020 [31]; Chen et al., 2024 [41]; Kirchberger et al., 2025 [53]; Kuo et al., 2023 [42]; Lin et al., 2023 [34]; Martinez-Hernandez et al., 2019 [43]; Mazumder et al., 2016 [44]; Netukova et al., 2021 [36]; Nishizawa et al., 2024 [37]; Parvaneh et al., 2017 [3]; Regterschot et al., 2014 [58]; Van Lummel et al., 2012 [47]; Wairagkar et al., 2022 [48]; Zakaria et al., 2015 [40]; Zijlstra et al., 2010 [50]
	>100 Hz	11	Al-Qaness et al., 2024 [52]; Amici et al., 2024 [10]; Atrsaei et al., 2020 [11]; Atrsaei et al., 2021 [29]; Marin et al., 2022 [35]; Parr et al., 2025 [38]; Pham et al., 2018 [46]; Salarian et al., 2010 [55]; Suglia et al., 2024 [54]; Wang et al., 2025 [49]; Witchel et al., 2018 [57]
	Not reported, partly reported, or dataset-dependent	7	Abraha et al., 2026 [51]; Al-Qaness et al., 2024 [52]; Atrsaei et al., 2020 [11]; Atrsaei et al., 2021 [29]; Braun et al., 2022 [30]; Caronni et al., 2018 [32]; Coulthard et al., 2015 [59]
Acquisition protocol	TUG/iTUG-based assessment	14	Abdollahi et al., 2024 [56]; Atrsaei et al., 2021 [29]; Braun et al., 2022 [30]; Buchman et al., 2020 [31]; Caronni et al., 2018 [32]; Coulthard et al., 2015 [59]; Lin et al., 2023 [34]; Netukova et al., 2021 [36]; Nishizawa et al., 2024 [37]; Regterschot et al., 2014 [58]; Salarian et al., 2010 [55]; Wang et al., 2024 [39]; Witchel et al., 2018 [57]; Zakaria et al., 2015 [40]
	Repeated STS/five-times STS/chair-rise protocols	10	Abdollahi et al., 2024 [56]; Atrsaei et al., 2020 [11]; Atrsaei et al., 2021 [29]; Braun et al., 2022 [30]; Kirchberger et al., 2025 [53]; Marin et al., 2022 [35]; Parr et al., 2025 [38]; Regterschot et al., 2014 [58]; Van Lummel et al., 2012 [47]; Zijlstra et al., 2010 [50]
	Custom postural-transition protocol	12	Amici et al., 2024 [10]; Bagala et al., 2013 [2]; Chen et al., 2024 [41]; Ganea et al., 2011 [33]; Kuo et al., 2023 [42]; Martinez-Hernandez et al., 2019 [43]; Mazumder et al., 2016 [44]; Najafi et al., 2003 [45]; Suglia et al., 2024 [54]; Wairagkar et al., 2022 [48]; Wang et al., 2025 [49]; Zijlstra et al., 2010 [50]
	Daily-life, free-living, or home-like monitoring	5	Atrsaei et al., 2020 [11]; Atrsaei et al., 2021 [29]; Najafi et al., 2003 [45]; Parvaneh et al., 2017 [3]; Pham et al., 2018 [46]
	HAR or public benchmark dataset protocol	4	Abraha et al., 2026 [51]; Al-Qaness et al., 2024 [52]; Kirchberger et al., 2025 [53]; Suglia et al., 2024 [54]

**Table 6:** Reported performance, success rate, and validation of IMU-based postural-transition methods across included studies.

**Study**	**IMU Configuration**	**Method/Purpose**	**Reference/Validation Standard**	**Performance Metric**	**Success Rate (%)**	**Reported Performance/Validation Result**
Abdollahi et al. (2024) [56]	8 IMUs	Threshold-based task start/end detection; clinical-test quantification	No independent PT gold standard	N/A	N/A	Method performance not evaluated; analyses focused on clinical/dual-task outcomes.
Abraha et al. (2026) [51]	1 body-worn inertial device; dataset-dependent	RF + XGBoost stacking ensemble for HAR/PT recognition	Dataset activity labels	Accuracy/F1	Overall accuracy 94.54–96.25%	KU-HAR 95.83%/95.00%; UCI-HAR 94.54%/94.44%; USC-HAD 96.25%/95.16%. Sit→Stand F1 0.90; Stand→Sit F1 0.89.
Al-Qaness et al. (2024) [52]	1 sensing device; dataset-dependent	PCNN–Transformer for multiclass HAR/fall recognition	Dataset class labels	F1/accuracy	Overall accuracy 84.80–98.01%	Transition-class F1: Sitting Down 94.44%; standing Up 93.75–95.24%; sit-to-stand 93.33%; stand-to-sit 84.51%. Overall multiclass accuracy 84.80–≈98%.
Amici et al. (2024) [10]	1 IMU	Threshold-based PT event detection and feature extraction	No external PT gold standard	N/A	N/A	No sensitivity/specificity/agreement metric for PT event detection was reported.
Atrsaei et al. (2020) [11]	Single-IMU algorithm; 4 body positions tested in Dataset A	CWT + peak detection + sigmoid-model PT selection	Video/observer PT events; Vicon for trunk tilt	PPV/sensitivity	Healthy: 98% PPV/95% sensitivity; disease: 89%/89%	Healthy: 98%/95%; participants with disease: 89%/89%. All 5×STS transitions detected correctly.
Atrsaei et al. (2021) [29]	3 IMUs in lab; 1 lower-back IMU free-living	Previously validated PT algorithms + decision-tree FOF classification	FES-I >19 for FOF status	AUC	N/A	FOF classification: combined lab + daily-life AUC 0.75; lab 0.68; daily-life 0.54. Not a PT-detection validation study.
Bagalà et al. (2013) [2]	1 body-fixed sensor	Rule-based LSSW event segmentation; clinical discrimination	Young vs. older group; repeated testing	Classification/AUC/ICC	≥92%	≥92% correct group classification; sensitivity/specificity 90–100%; AUC ≥0.98; ICC 0.85–0.95. Not PT-detection validation.
Braun et al. (2022) [30]	3 IMUs	Mobility/STS quantification; regression analyses	No concurrent PT reference	N/A	N/A	No PT detection/classification, reliability, validity, or agreement metric reported.
Buchman et al. (2020) [31]	1 body-worn sensor	Automated mobility quantification using previously validated formulas	No external PT reference	N/A	N/A	No PT detection/classification performance metric; study focused on prognostic associations.
Caronni et al. (2018) [32]	1 IMU	Manual TUG phase segmentation + PLSR criterion-validity analysis	Mini-BESTest	R^2^/RMSEP	N/A	Criterion validity: R^2^ = 0.41, RMSEP ≈ 11%; turning-only model R^2^ = 0.43, RMSEP ≈ 10.5%. Not PT-detection success.
Chen et al. (2024) [41]	2 IMUs	Thresholding + CNN–BiLSTM–Attention for five STS phases	Force-plate vertical GRF	Accuracy/precision/recall/F1	99.5%	99.5% for all four metrics.
Coulthard et al. (2015) [59]	6 IMUs	Commercial iTUG segmentation; subtask quantification	Manual stopwatch for total TUG only	R^2^ (task-level agreement)	NR (PT-specific)	Total TUG R^2^ = 0.95 vs. stopwatch; ≈2 s overestimation. PT-specific segmentation accuracy NR.
Ganea et al. (2011) [33]	1 inertial sensor	Rule-based trunk-tilt segmentation; frailty discrimination	POMA/group status	Sensitivity/specificity/AUC	92% sensitivity/88% specificity	SiSt smoothness: Sensitivity 0.92, specificity 0.88, AUC 0.96 for frail vs. healthy. Not PT-detection validation.
Kirchberger et al. (2025) [53]	3 sensors worn; single-location models compared	Random Forest activity classification	Investigator activity labels	Accuracy/weighted F1	84.6% overall; STS-specific NR	Best ankle accelerometer + gyroscope model: accuracy 84.6%, weighted F1 ≈ 84.1%; wrist/thigh 72.4–76.8%. STS-specific accuracy NR.
Kuo et al. (2023) [42]	4 IMUs	LSTM/FNN posture-state and transition-time recognition	Manual labels; Vicon for selected timing comparison	Accuracy/timing error	94.44% at 100 Hz	LSTM ≈ 91% at 25 Hz and ≈95% at 100 Hz; 100 Hz posture identification 94.44%; transition-time error 0.17 s.
Lin et al. (2023) [34]	10 IMUs	TUG segmentation + ensemble PD vs. ET classification	Clinical PD/ET diagnosis	Accuracy/sensitivity/specificity/AUC	75.8%	Independent test: 75.8%/80.0%/69.2%/0.823; LOOCV AUC 0.912. Diagnostic, not PT-detection performance.
Marin et al. (2022) [35]	1 sensor	PCA/k-means STS scoring and PD discrimination	MDS-UPDRS III/group status	Sensitivity/specificity/R^2^	40% sensitivity/96% specificity	PD lower-cluster classification: Sensitivity 0.40, specificity 0.96; a-/f-score association R^2^ = 0.28/0.24. Not PT-detection success.
Martinez-Hernandez et al. (2019) [43]	1 wearable sensor	Bayesian sequential SiSt/StSi state/phase recognition	Labeled activity/phase data	Accuracy/response time	100%	100% identification accuracy; mean response time 50 ms.
Mazumder et al. (2016) [44]	2 IMUs + pressure-sensor insole	Dendrogram-based SVM activity/PT classification	Protocol activity labels	Accuracy	95.2% overall; SiSt 97%; StSi 100%	Overall 95.2%; sit-to-stand 97%; stand-to-sit 100%.
Najafi et al. (2003) [45]	1 chest kinematic sensor	DWT + kinematic rule model for PT/activity detection	Vicon/observer reference	Sensitivity/specificity/accuracy	99% overall PT sensitivity	Overall PT sensitivity 99%; SiSt 93%/82%; StSi 82%/94%; transfers/rolling >99% accuracy.
Netuková et al. (2021) [36]	1 gyro-accelerometer	Threshold-based STS detection + continuous-curve reliability analysis	No external PT reference	ICC	N/A	Pitch angular velocity ICC 0.67 controls/0.62 PD; AP acceleration 0.69/0.45. Reliability, not detection success.
Nishizawa et al. (2024) [37]	2 IMUs	TUG feature extraction + logistic-regression LS discrimination	Locomotive Syndrome stage	Accuracy/sensitivity/specificity/AUC	72.2%	Correct classification 72.2%; sensitivity 55–60%; specificity 80.7–84.2%; AUC 0.70–0.75. Not PT-detection validation.
Parr et al. (2025) [38]	2 IMUs	Threshold/slope-based STS segmentation and trunk-control quantification	No external PT reference	N/A	N/A	Custom segmentation used, but no detection/classification validation, reliability, or agreement metric reported.
Parvaneh et al. (2017) [3]	1 wearable sensor	Previously validated PAMSys PT counting + frailty association	Fried frailty status	NR in current study	NR	No in-study PT-detection validation; current study focused on associations between daily PT behavior and frailty.
Pham et al. (2018) [46]	1 lower-back IMU	DWT + 6DOF fusion + vertical-displacement PT detection	Video; two raters	Detection accuracy/direction accuracy/ICC	82% PD no dysk.; 47% PD dysk.; 85% older adults; direction 98%	Detection: 82% PD without dyskinesia, 47% PD with dyskinesia, 85% older adults; PT direction 98%; inter-rater ICC 0.99.
Regterschot et al. (2014) [58]	1 hybrid motion sensor	Sensor-based STS power quantification; test–retest study	Repeated assessment	Reliability	N/A	Peak-power reliability reported as excellent; PT detection/classification performance was not evaluated.
Salarian et al. (2010) [55]	7 inertial sensors	Automatic iTUG segmentation and mobility quantification	Video/stopwatch comparators	ICC/model fit	NR	PT-detection accuracy NR. STS ICCs: 0.04–0.43; turn-to-sit duration/ROM ICCs 0.84/0.77; turn-model fit R^2^ = 0.9989.
Suglia et al. (2024) [54]	4 IMUs acquired; 2-, 3-, and 4-sensor combinations tested	Multi-branch 1D-CNN continuous HAR	Manual labels	Accuracy by sensor configuration	2 IMUs 95.00%; 3 IMUs 96.69%; 4 IMUs 96.06%	Best grouped-window accuracy 96.69% with 3 IMUs (RW + RP + LP); 4 IMUs 96.06%; direct 2-/3-/4-sensor comparison.
Van Lummel et al. (2012) [47]	1 body-fixed inertial sensor	Feature-based seat-off/seat-on event detection	Synchronized chair switches	Mean absolute timing error	N/A	Seat-off 51 ms (mean error 0 ms); seat-on 127 ms (mean error −3 ms), leave-one-out validation.
Wairagkar et al. (2022) [48]	2 IMUs	Kinematic model + EKF + unsupervised PT/state classification	Codamotion motion capture in young healthy adults	Classification accuracy	98.67% young; 94.20% older; 91.41% PD	98.67% young healthy; 94.20% older healthy; 91.41% Parkinson’s disease.
Wang et al. (2024) [39]	1 IMU + depth camera	TUG feature extraction + fall-risk discrimination	Berg Balance Scale grouping	AUC	NR	Combined IMU + depth-camera AUC 0.882; PT-specific classification accuracy NR.
Wang et al. (2025) [49]	7 IMUs + 16-channel sEMG	iTransformer-DTL for STS phase recognition and joint-angle prediction	Kinematic event definitions	Accuracy/F1/recall/MAE	99.22 ± 0.76% overall	Healthy: 99.42% accuracy, F1 98.71%, recall 98.89%; stroke: 99.01%, 97.93%, 98.11%; overall accuracy 99.22 ± 0.76%.
Witchel et al. (2018) [57]	3 IMU nodes	Transparent STS/StSi feature extraction + MS discrimination	Clinical group status	Correct classification/ICC	79.82%	7-feature model correct prediction 79.82%; selected STS/StSi ICCs 0.61–0.85. Not PT-detection success.
Zakaria et al. (2015) [40]	1 waist IMU	Rule-based TUG phase segmentation + k-NN fall-risk classification	13.5 s TUG threshold for fall-risk grouping	NR	NR	No numerical accuracy/sensitivity/specificity reported for the proposed k-NN classifier.
Zijlstra et al. (2010) [50]	3 trunk IMU nodes (+ 1 synchronization node)	Sensor-derived STS vertical acceleration/power estimation	Force plate	Correlation	N/A	Peak-power correlation vs. force plate R = 0.92–0.99 in young adults and R = 0.93–0.96 in older adults, depending on sensor location.

Note: Performance values are reported as provided by the original studies. Metrics were not converted into a common success category because analytical purposes, reference standards, and performance measures differed substantially. Success rate (%) indicates a percentage-based measure of correct detection or classification when reported by the original study; AUC, ICC, R^2^, MAE, correlation coefficients, and other non-percentage metrics were not converted into success rates. N/A = not applicable; NR = not reported. Abbreviations: 1D-CNN = one-dimensional convolutional neural network; 5×STS = Five-Times Sit-to-Stand; 6DOF = six degrees of freedom; AP = anteroposterior; AUC = area under the receiver operating characteristic curve; BiLSTM = bidirectional long short-term memory; CNN = convolutional neural network; CWT = continuous wavelet transform; DTL = dual-task learning; DWT = discrete wavelet transform; EKF = extended Kalman filter; ET = Essential Tremor; F1 = F1-score (harmonic mean of precision and recall); FES-I = Falls Efficacy Scale–International; FNN = feedforward neural network; FOF = Fear of Falling; GRF = Ground Reaction Force; HAR = Human Activity Recognition; Hz = hertz; ICC = intraclass correlation coefficient; IMU = Inertial Measurement Unit; iTUG = Instrumented Timed Up and Go; k-NN = k-nearest neighbors; KU-HAR = Khulna University Human-Activity-Recognition dataset; LOOCV = leave-one-out cross-validation; LP = left pelvis; LS = Locomotive Syndrome; LSSW = lie-to-sit-to-stand-to-walk; LSTM = long short-term memory; MAE = mean absolute error; MDS-UPDRS III = Movement Disorder Society-sponsored revision of the Unified Parkinson’s Disease Rating Scale, Part III; Mini-BESTest = Mini-Balance Evaluation Systems Test; MS = multiple sclerosis; ms = milliseconds; N/A = not applicable; NR = not reported; PAMSys = physical activity monitoring system; PCA = principal component analysis; PCNN = parallel convolutional neural network; PD = Parkinson’s disease; PLSR = partial least squares regression; POMA = Performance-Oriented Mobility Assessment; PPV = positive predictive value; PT = postural transition; R = correlation coefficient; R^2^ = coefficient of determination; RF = Random Forest; RMSEP = Root Mean Square error of prediction; ROM = Range of Motion; RP = right pelvis; RW = right wrist; sEMG = surface electromyography; SiSt = sit-to-stand; StSi = stand-to-sit; STS = sit-to-stand; SVM = support vector machine; TUG = Timed Up and Go; UCI-HAR = University of California Irvine Human Activity Recognition dataset; USC-HAD = University of Southern California Human Activity Dataset; and XGBoost = Extreme Gradient Boosting.

**Table 7:** Summary of IMU-derived outcome domains and clinical or functional interpretation of postural-transition metrics.

	**Domain**	**Main IMU-Derived Outcomes**	**Clinical or Functional** **Interpretation**	**Studies, n [Refs]**
Outcome domains	Temporal parameters	TUG time; STS/STS duration; transition and phase duration; seat-off/seat-on timing; completion time; transition counts; daily-state duration; response/inference time.	Quantify task timing, event structure, and daily-life transition frequency; clinically intuitive but limited for movement quality.	20 studies [2,3,11,29,30,31,32,33,37,39,40,42,45,46,47,53,55,56,57,58]
	Kinematic parameters	Trunk/segment acceleration; angular velocity/displacement; trunk tilt; pitch/yaw/roll angles; ROM; vertical displacement; gait, turning, and joint kinematics.	Describe how transitions are performed, including segmental control, trunk/lower-limb movement, and phase-specific patterns.	22 studies [2,11,30,31,34,35,36,37,39,40,41,44,45,47,48,49,50,55,56,57,58,59]
	Movement-quality parameters	Jerk, RMS jerk, smoothness, fluency, fractal dimension, wavelet energy, movement irregularity, acceleration variability, trunk movement variation.	Capture movement quality beyond duration, potentially reflecting compensations, motor-control deficits, and inefficient execution.	8 studies [2,10,30,34,38,39,57,58]
	Force-, power-, or energy-related outcomes	Peak power; force-related trunk estimates; vertical force or power from acceleration; kinetic energy; GRF-based phase labeling.	Reflect mechanical output, force production, and energetic aspects of STS, especially when linked to biomechanical models or references.	7 studies [11,29,35,41,49,50,58]
	Classification and performance metrics	Accuracy, precision, recall, F1-score, sensitivity, specificity, AUC, confusion matrices, response time, loss, inference time.	Support detection, classification, and HAR; not necessarily evidence of valid biomechanical onset/offset or subphase segmentation.	11 studies [40,41,42,43,44,48,49,51,52,53,54]
	**Clinical population**	**Main IMU-derived outcomes**	**Clinical or functional interpretation**	**Studies, n [refs]**
Clinical or functional interpretation	Stroke and mixed neurorehabilitation	Instrumented TUG, STS, walking, and balance-related measures; dual-task-related changes.	Used to characterize slower task performance and functional changes in stroke and mixed neurorehabilitation cohorts.	2 studies [10,56]
	Parkinson’s disease	Transition duration; peak angular velocity; trunk/limb kinematics; turn-to-sit behavior; phase-recognition and daily-life mobility features.	Used to characterize Fear of Falling, early PD versus Essential Tremor, STS performance, medication/dyskinesia-related features, and phase abnormalities.	8 studies [29,32,34,35,36,46,48,55]
	Multiple sclerosis	Thigh-derived angular-velocity features during STS and stand-to-sit.	Clinically informative and more discriminatory than some purely temporal measures for mobility impairment in MS.	1 study [57]
	Older adults, frailty, and fall-risk populations	Transition duration, smoothness, daily transition number, cautious sitting, TUG abnormalities, power-related and health-outcome-associated features.	Used to describe frailty/fall-risk abnormalities, daily-life mobility behavior, and associations with adverse health outcomes.	8 studies [2,3,31,33,39,40,50,58]
	Other clinical applications	PT metrics in geriatric heart failure, Locomotive Syndrome, transtibial amputation with/without low back pain, acute-care patients, and mixed rehabilitation.	Shows use across heterogeneous clinical contexts, although interpretation is often population-specific and not always externally validated.	5 studies [10,30,37,38,53]

Abbreviations: AUC: Area Under the Curve; GRF: Ground Reaction Force; HAR: Human Activity Recognition; IMU: Inertial Measurement Unit; MS: multiple sclerosis; PD: Parkinson’s Disease; PT: postural transition; RMS: Root Mean Square; ROM: Range of Motion; STS: sit-to-Stand; TUG: Timed Up and Go. Note: Categories are not mutually exclusive because several studies reported outcomes belonging to more than one domain or applied PT metrics to more than one clinical or functional purpose.

## Data Availability

The dataset analyzed for this study is available at the Zenodo repository: https://doi.org/10.5281/zenodo.21353814.

## Supplementary Materials

The following supporting information can be downloaded at: https://www.mdpi.com/article/10.3390/s26216808/s1, Table S1: Operational criteria for assigning overall methodological judgments; Table S2: Bibliographic details of the studies included in the systematic review; Table S3: Full dataset of extracted data.

## Abbreviations

AUC	Area Under the Receiver Operating Characteristic Curve
BiLSTM	Bidirectional Long Short-Term Memory
CNN	Convolutional Neural Network
COSMIN	Consensus-based Standards for the Selection of Health Measurement Instruments
DOI	Digital Object Identifier
HAR	Human Activity Recognition
ICC	Intraclass Correlation Coefficient
IMU	Inertial Measurement Unit
JBI	Joanna Briggs Institute
LSTM	Long Short-Term Memory
PICO	Population, Intervention, Comparison, Outcome
PRISMA	Preferred Reporting Items for Systematic Reviews and Meta-Analyses
PT	Postural Transition
QUIPS	Quality In Prognosis Studies
RMS	Root Mean Square
SEM	Standard Error of Measurement
SVM	Support Vector Machine
TUG	Timed Up and Go
