# Supplementary material for: IMU-Based Analysis of Postural Transitions: A Systematic Review
Source: Sensors (Basel). 2026 Oct 28;26(21):6808. doi: 10.3390/s26216808 (PMC13765966; doi:10.3390/s26216808)
Supplement: Supplementary file 1 [file sensors-26-06808-s001.zip › Supplementary Material S1_Table S1.pdf]

Supplementary Material S1_Table S1. Operational criteria for assigning overall methodological judgments

Overall judgement	Operational meaning
**Some concerns**	Minor or isolated limitations; findings remain reasonably interpretable.
**Moderate concerns**	Relevant limitations present, but not enough to seriously compromise the main findings.
**Moderate-to-high concerns**	Multiple or important limitations reduce confidence in the findings.
**High concerns**	Major methodological flaws substantially limit validity, interpretability, or applicability.
