# Supplementary material for: IMU-Based Analysis of Postural Transitions: A Systematic Review
Source: Sensors (Basel). 2026 Oct 28;26(21):6808. doi: 10.3390/s26216808 (PMC13765966; doi:10.3390/s26216808)
Supplement: Supplementary file 1 [file sensors-26-06808-s001.zip › Supplementary Material S2_Table S2.pdf]

Supplementary Material S2 - Table S2. Bibliographic details of the studies included in the systematic review.

Author/year	Title	DOI
Abdollahi et al., 2024	Dual Tasking Affects the Outcomes of Instrumented Timed up and Go, Sit-to-Stand, Balance, and 10-Meter Walk Tests in Stroke Survivors	10.3390/s24102996
Abraha et al., 2026	Stacking ensemble-based predictive framework for human activity and postural transition recognition using wearable sensors	10.1016/j.engappai.2026.114782
Al-qaness et al., 2024	Human activity recognition and fall detection using convolutional neural network and transformer-based architecture	10.1016/j.bspc.2024.106412
Amici et al., 2024	Kinematic IMU-Based Assessment of Postural Transitions: A Preliminary Application in Clinical Context	10.3390/app14167011
Atrsaei et al., 2020	Postural transitions detection and characterization in healthy and patient populations using a single waist sensor	10.1186/s12984-020-00692-4
Atrsaei et al., 2021	Effect of Fear of Falling on Mobility Measured During Lab and Daily Activity Assessments in Parkinson's Disease	10.3389/fnagi.2021.722830
Bagalà et al., 2013	Quantitative description of the lie-to-sit-to-stand-to-walk transfer by a single body-fixed sensor	10.1109/TNSRE.2012.2230189
Braun et al., 2022	Association between heart failure severity and mobility in geriatric patients: an in-clinic study with wearable sensors	10.11909/j.issn.1671-5411.2022.09.010
Buchman et al., 2020	Different Combinations of Mobility Metrics Derived From a Wearable Sensor Are Associated With Distinct Health Outcomes in Older Adults	10.1093/gerona/glz160
Caronni et al., 2018	Criterion validity of the instrumented Timed Up and Go test: A partial least square regression study	10.1016/j.gaitpost.2018.01.015
Chen et al., 2024	A Novel CNN-BiLSTM Ensemble Model With Attention Mechanism for Sit-to-Stand Phase Identification Using Wearable Inertial Sensors	10.1109/TNSRE.2024.3366907
Coulthard et al., 2015	Evaluation of an inertial sensor system for analysis of timed-up-and-go under dual-task demands	10.1016/j.gaitpost.2015.03.009
Ganea et al., 2011	Multi-parametric evaluation of sit-to-stand and stand-to-sit transitions in elderly people	10.1016/j.medengphy.2011.04.015
Kirchberger et al., 2025	An explorative study on movement detection using wearable sensors in acute care hospital patients	10.1038/s41598-025-04340-6
Kuo et al., 2023	Human Posture Transition-Time Detection Based upon Inertial Measurement Unit and Long Short-Term Memory Neural Networks	10.3390/biomimetics8060471

Author/year	Title	DOI
Lin et al., 2023	Wearable sensor-based gait analysis to discriminate early Parkinson’s disease from essential tremor	10.1007/s00415-023-11577-6
Marin et al., 2022	Scoring the Sit-to-Stand Performance of Parkinson’s Patients with a Single Wearable Sensor	10.3390/s22218340
Martinez-Hernandez et al., 2019	Probabilistic identification of sit-to-stand and stand-to-sit with a wearable sensor	10.1016/j.patrec.2018.03.020
Mazumder et al., 2016	Ambulatory activity classification with dendogram-based support vector machine: Application in lower-limb active exoskeleton	10.1016/j.gaitpost.2016.08.010
Najafi et al., 2003	Ambulatory system for human motion analysis using a kinematic sensor: monitoring of daily physical activity in the elderly	10.1109/TBME.2003.812189
Netuková et al., 2021	The timed up & go test sit-to-stand transition: Which signals measured by inertial sensors are a viable route for continuous analysis?	10.1016/j.gaitpost.2020.11.006
Nishizawa et al., 2024	Turning and sitting movements during timed up and go tests predict deterioration of physical function in middle-aged adults	10.1016/j.gaitpost.2023.12.020
Parr et al., 2025	Low back pain influences medial-lateral trunk movement variations during sit-to-stand tasks in persons with transtibial amputation	10.1016/j.clinbiomech.2025.106623
Parvaneh et al., 2017	Postural Transitions during Activities of Daily Living Could Identify Frailty Status: Application of Wearable Technology to Identify Frailty during Unsupervised Condition	10.1159/000460292
Pham et al., 2018	Validation of a Lower Back “Wearable”-Based Sit-to-Stand and Stand-to-Sit Algorithm for Patients With Parkinson’s Disease and Older Adults in a Home-Like Environment	10.3389/fneur.2018.00652
Regterschot et al., 2014	Test-retest reliability of sensor-based sit-to-stand measures in young and older adults	10.1016/j.gaitpost.2014.03.193
Salarian et al., 2010	iTUG, a sensitive and reliable measure of mobility	10.1109/TNSRE.2010.2047606
Suglia et al., 2024	A Novel Framework Based on Deep Learning Architecture for Continuous Human Activity Recognition with Inertial Sensors	10.3390/s24072199
Van Lummel et al., 2012	Validation of seat-off and seat-on in repeated sit-to-stand movements using a single-body-fixed sensor	10.1088/0967-3334/33/11/1855
Wairagkar et al., 2022	A novel approach for modelling and classifying sit-to-stand kinematics using inertial sensors	10.1371/journal.pone.0264126
Wang et al., 2024	Identifying sensors-based parameters associated with fall risk in community-dwelling older adults: an investigation and interpretation of discriminatory parameters	10.1186/s12877-024-04723-w

Author/year	Title	DOI
Wang et al., 2025	A Dual-Task Improved Transformer Framework for Decoding Lower Limb Sit-to-Stand Movement from sEMG and IMU Data †	10.3390/machines13100953
Witchel et al., 2018	Thigh-Derived Inertial Sensor Metrics to Assess the Sit-to-Stand and Stand-to-Sit Transitions in the Timed Up and Go (TUG) Task for Quantifying Mobility Impairment in Multiple Sclerosis	10.3389/fneur.2018.00684
Zakaria et al., 2015	Quantitative analysis of fall risk using TUG test	10.1080/10255842.2013.805211
Zijlstra et al., 2010	A body-fixed-sensor-based analysis of power during sit-to-stand movements	10.1016/j.gaitpost.2009.11.003

Abbreviations: DOI, Digital Object Identifier.
